# Supplementary material for: TET1-mediated DNA hydroxymethylation regulates adult remyelination in mice
Source: Nat Commun. 2021 Jun 7;12:3359. doi: 10.1038/s41467-021-23735-3 (PMC8185117; doi:10.1038/s41467-021-23735-3)
Supplement: Supplementary file 1 — Supplementary information [file 41467_2021_23735_MOESM1_ESM.pdf]

Supplementary Information

TET1-mediated DNA hydroxymethylation regulates adult remyelination in mice

Sarah Moyon[1,#], Rebecca Frawley[1], Damien Marechal[1], Dennis Huang[1], Katy LH Marshall-Phelps[3], Linde Kegel[3], Sunniva MK Bøstrand[3], Boguslawa Sadowski[4,5,6], Yong-Hui Jiang[7], David Lyons[3], Wiebke Möbius[4,5,6] and Patrizia Casaccia[1,2,#]

Supplementary Table 1

Gene symbol	log2FoldChange	pvalue	padj	Gene symbol	log2FoldChange	pvalue	padj
Aars	0.68237575	4.27E-05	0.000777211	*Ncapg2*	0.858787848	0.006408161	0.037514234
Aatk	0.639773595	0.006079162	0.036083692	*Ncdn*	0.634631605	8.87E-05	0.001414309
Abat	0.729677401	0.000959102	0.009147281	*Nckap1*	0.986642242	0.000205239	0.002735984
Abca1	0.401621896	0.009088433	0.048393986	*Ncoa1*	0.823566028	0.000843447	0.008226872
Abca2	1.106502259	3.46E-08	1.89E-06	*Ncoa2*	1.311660835	6.37E-05	0.001078862
Abca3	1.252498365	0.000308798	0.003765942	*Ncoa6*	0.838796835	0.003762466	0.025149187
Abca5	0.988271233	1.31E-06	4.39E-05	*Ndc1*	0.632276737	0.00273002	0.019810197
Abca6	1.297976339	1.89E-07	8.36E-06	*Nde1*	0.71536838	0.001848296	0.014867382
Abca8a	1.293414695	6.60E-05	0.001101518	*Ndnf*	1.283879034	0.008461944	0.046083584
Abcb7	0.922066061	1.24E-05	0.000281567	*Ndrg2*	1.873860866	7.08E-38	7.04E-35
Abcc1	1.022916769	1.66E-07	7.44E-06	*Ndst1*	0.610970482	0.002025252	0.015872033
Abhd13	0.843946737	4.80E-07	1.86E-05	*Necap1*	0.539788596	0.004132855	0.027022952
Abi1	0.444681327	0.004305091	0.027888666	*Nedd4*	0.720318626	0.00037426	0.004339069
Abl1	0.837857147	0.00173327	0.014189696	*Negr1*	0.851314694	0.000150813	0.002143477
Abtb2	0.936792788	2.00E-05	0.000421587	*Nek4*	0.573725024	0.00352954	0.023938977
Acaca	0.842444448	0.004011233	0.02640123	*Nek7*	0.951551256	1.61E-06	5.26E-05
Acadsb	0.641840613	0.000217257	0.002849969	*Nek9*	0.734862585	0.000547268	0.005867843
Acap2	1.064863584	4.33E-06	0.000120116	*Neo1*	1.297377968	1.72E-08	1.03E-06
Acap3	0.775698737	0.001199887	0.010795553	*Neto2*	1.345885991	2.32E-07	9.96E-06
Acbd3	0.678876049	0.001313735	0.011566616	*Nf2*	1.189015279	0.000177208	0.00243619
Acbd5	0.771313307	5.45E-05	0.000952082	*Nfatc3*	0.977436061	3.44E-06	9.83E-05
Acly	0.708646261	0.000227272	0.002956725	*Nfe2l1*	1.101222244	4.28E-05	0.000777475
Acot11	1.336818803	2.72E-05	0.000537711	*Nfe2l3*	0.6533215	0.000488142	0.005386656
Acox1	0.726077104	1.62E-05	0.000351997	*Nhlrc1*	0.643972888	0.005667628	0.034217558
Acp2	0.710407954	0.001031231	0.009678931	*Nhlrc3*	0.968574702	0.003735376	0.025012332
Acsl1	0.73133938	0.001357511	0.011814471	*Nipa1*	0.529398829	0.008050119	0.044533626
Acsl4	0.892390266	0.000154063	0.002184735	*Nipal3*	0.441216976	0.003901615	0.025803148
Acvr2a	1.158951956	0.000232419	0.003007129	*Nipal4*	0.560338243	0.002259467	0.017173587
Adam10	1.154414075	2.09E-06	6.52E-05	*Nisch*	0.657294289	0.001738718	0.014221978
Adam17	1.308329423	2.43E-08	1.41E-06	*Nkd2*	4.253863325	0.001122855	0.010308558
Adam19	0.709068038	0.005161124	0.031868904	*Nlgn1*	0.731453598	0.001578235	0.013271073
Adam22	0.921035504	0.000671308	0.006870457	*Nlgn3*	0.972384476	0.000662791	0.0068017
Adam9	1.041031507	2.41E-06	7.36E-05	*Noc3l*	0.781469049	0.000111737	0.001682996
Adamts1	0.800719463	0.000197443	0.002658314	*Nod2*	4.59312434	0.000263244	0.003317398
Adamts2	0.542475514	0.006566245	0.038226585	*Nol4l*	1.031345298	9.22E-05	0.001444647
Adamts4	0.545261745	0.008001415	0.04427715	*Nol6*	0.67127542	0.000284716	0.003524546
Adcy5	0.794330819	5.47E-08	2.83E-06	*Nol9*	0.674962293	0.001699989	0.01400213
Add1	0.698623695	0.000159598	0.002241368	*Nom1*	0.530896571	0.001767544	0.014401673
Add3	0.815322325	0.000339484	0.004036019	*Nomo1*	0.76899541	0.000554691	0.005920566
Adgra2	1.20796883	6.54E-05	0.001094433	*Nono*	0.517243477	0.003728008	0.024980693
Adgra3	1.061019575	0.000360913	0.004219129	*Npat*	0.778185515	6.38E-05	0.00107914
Adgrf5	6.626847971	1.52E-05	0.000334578	*Npnt*	3.457552882	1.07E-05	0.000251274
Adgrg2	1.246742444	6.34E-07	2.34E-05	*Npy*	4.637143107	0.003242621	0.022518465
Adgrl3	0.630743906	0.004057955	0.026625309	*Nr1d2*	0.737167211	0.000575552	0.006081421
Adgrl4	6.668585928	0.00012108	0.001799346	*Nr2c2*	0.610804934	0.000919462	0.008822636
Adi1	2.467233104	3.37E-32	2.55E-29	*Nsg1*	1.900943736	2.80E-23	1.08E-20
Aebp2	0.701780858	2.53E-05	0.000507767	*Nub1*	0.593627121	0.000858056	0.008343534
Afap1	0.642315521	0.000247624	0.003164867	*Nudt4*	0.609699085	0.004390423	0.02828024
Aff4	0.932136181	0.000274533	0.003434473	*Numa1*	1.113589815	0.001806342	0.014629511
Agfg1	0.924607333	1.34E-05	0.000301239	*Nup153*	0.692818173	0.001211928	0.010878007
Ago1	0.545660387	0.005453439	0.03321081	*Nup160*	0.959914197	0.000124803	0.00184019
Agpat3	0.494966607	0.005655931	0.034157846	*Nup188*	0.690138447	0.000239404	0.003080632
Agps	0.890005615	7.33E-05	0.001200976	*Nup205*	1.0018033	0.001122406	0.010308558
Agtpbp1	0.550374685	0.002444384	0.018241007	*Nup98*	0.686848879	0.003497121	0.023770612
Ahctf1	0.938728538	0.002040401	0.015957678	*Ocel1*	0.831168323	2.17E-06	6.75E-05
Aifm2	0.522734802	0.004900424	0.030703587	*Oga*	0.728139991	0.001155721	0.010528475
Ak4	1.936791159	0.001353948	0.011802372	*Oip5os1*	0.882288986	2.26E-05	0.000467515
Akap1	0.682424065	8.94E-05	0.001423031	*Olfml2b*	0.779892088	0.002052347	0.016004754
Akap6	1.027295806	2.01E-05	0.000424085	*Opa1*	1.110571498	8.66E-07	3.08E-05
Akt2	0.520407996	0.002804662	0.020196772	*Opa3*	0.423148474	0.00878044	0.047246417
Alcam	0.927882494	5.67E-06	0.000150675	*Oplah*	0.482744483	0.002474962	0.018378655
Aldh6a1	0.821669307	7.97E-05	0.001289565	*Ormdl3*	0.492993408	0.001852551	0.014879017
Alg11	0.685901633	0.000839474	0.00820081	*Osbpl11*	0.827327171	0.001089606	0.010076721
Als2	0.954206451	0.000161119	0.002254348	*Osbpl2*	0.450499831	0.000953891	0.009105828

Alyref2	1.206325528	0.001318293	0.011590277	*Osbpl8*	0.817886339	0.000340331	0.004043547
Ambra1	0.972946824	6.97E-05	0.001153607	*Ostm1*	0.563458129	0.000280809	0.003492192
Amd2	2.337041586	1.12E-07	5.21E-06	*Otud4*	0.95067507	0.000381932	0.004412996
Amigo1	1.273097113	6.90E-11	6.83E-09	*Otud6b*	0.664905685	0.000585811	0.006169132
Amotl2	0.961442248	6.99E-05	0.001154555	*Otud7b*	0.52355672	0.00310206	0.021814825
Amph	0.667123758	0.00040621	0.00464435	*Pabpc5*	1.060889339	0.001331232	0.011682341
Anapc1	0.627201729	0.001843843	0.014837875	*Pacs2*	0.949748973	0.000175633	0.0024163
Ank	0.569859789	0.000978871	0.009274986	*Pacsin1*	1.046869098	0.00035458	0.004163293
Ank2	0.87814521	0.002643159	0.019313348	*Pacsin2*	0.754424224	0.003721605	0.024964335
Ankfy1	1.06377174	0.000210711	0.002779529	*Paip2b*	0.783099823	0.00055601	0.005931301
Ankib1	1.113240469	4.67E-07	1.82E-05	*Pak1*	0.527574024	0.000110576	0.001673509
Ankle2	0.726034025	0.000578833	0.006105445	*Pak2*	0.658685542	0.000643336	0.00664898
Ankrd13a	0.87508905	3.39E-05	0.000642975	*Pam*	1.692002809	6.03E-07	2.25E-05
Ankrd13c	0.689241469	0.000220419	0.002881451	*Pank3*	1.180474132	1.13E-07	5.28E-06
Ankrd44	0.70926688	0.004155068	0.027158802	*Papola*	0.713337707	0.000202455	0.002706505
Ankrd50	1.071467162	3.31E-05	0.000632141	*Papolg*	0.862816503	1.15E-06	3.94E-05
Anks1	1.358117469	1.05E-07	4.93E-06	*Pappa2*	6.866887379	3.42E-06	9.80E-05
Anln	0.925952802	0.000717306	0.007254812	*Pard6b*	6.782690197	3.35E-05	0.000638044
Anp32e	0.801881009	5.21E-05	0.000918938	*Parp11*	0.539814741	0.003634486	0.02451041
Ap1b1	0.833883395	2.87E-06	8.48E-05	*Patl1*	0.707325135	0.000665379	0.00682456
Ap1g1	0.863625972	8.83E-05	0.001408963	*Pcdh1*	1.075067918	5.08E-06	0.000137789
Ap2a2	0.539864688	0.001196958	0.010782652	*Pcdh10*	0.528905588	0.003244066	0.022520231
Ap3m1	0.619722674	7.47E-05	0.001218276	*Pcdhb11*	1.477024032	0.000455398	0.005085695
Ap3m2	0.836145027	0.000560905	0.005970037	*Pcdhb15*	1.363318692	0.000325346	0.003907249
Ap5m1	0.832075069	9.84E-06	0.000235952	*Pcdhb16*	1.147571393	0.000698037	0.007086466
Apba1	1.296406697	4.60E-05	0.000828214	*Pcdhb2*	1.92888439	0.0034953	0.023766782
Apbb2	0.624244883	0.001672854	0.013808713	*Pcdhb20*	1.4837456	6.32E-09	4.22E-07
Apc	0.849051373	0.008333057	0.04558793	*Pcdhb22*	1.396262062	0.000209858	0.002773483
Apcdd1	1.421372626	4.28E-07	1.69E-05	*Pcdhb9*	0.788991423	0.003242596	0.022518465
Aplp2	0.91742846	1.65E-05	0.000358032	*Pcdhga12*	0.81529668	0.00598194	0.035701792
App	0.570601286	0.00279483	0.020148995	*Pcdhga4*	2.990021942	2.91E-14	4.63E-12
Appl1	0.659990685	0.000877716	0.008499731	*Pcdhga5*	1.322900107	4.72E-05	0.000848148
Aqp1	4.946915056	4.88E-08	2.57E-06	*Pcdhga6*	1.400246933	3.22E-05	0.000616264
Arap2	0.529089955	0.009236904	0.04891807	*Pcdhga8*	1.28001057	1.46E-07	6.61E-06
Arcn1	0.579772283	0.006561993	0.038213601	*Pcdhga9*	1.501999251	2.61E-05	0.000521271
Arel1	0.976891067	1.64E-08	9.93E-07	*Pcdhgb1*	1.63360407	5.12E-06	0.0001383
Arfgef1	0.782118275	0.000134806	0.001958675	*Pcdhgb4*	0.650350835	0.008347225	0.045620851
Arfgef2	1.124110118	0.001714297	0.014064826	*Pcdhgb8*	3.904868065	2.17E-10	2.01E-08
Arhgap12	0.714806332	2.16E-05	0.000449667	*Pcgf3*	0.794422765	2.41E-06	7.36E-05
Arhgap19	1.01240255	0.003221017	0.022417851	*Pcid2*	0.46806497	0.002488034	0.018450885
Arhgap21	1.264422481	0.001318875	0.011590277	*Pcif1*	0.493791435	0.000338849	0.004036019
Arhgap27	2.679329576	0.001669751	0.01378912	*Pcm1*	0.87787796	3.92E-08	2.11E-06
Arhgap31	1.158551776	1.84E-06	5.90E-05	*Pcmtd1*	0.796674784	0.000315914	0.003825577
Arhgap35	0.688623275	0.004812603	0.0303647	*Pcmtd2*	0.622551161	6.21E-06	0.000162523
Arhgap5	0.646024003	0.000796274	0.007864138	*Pcnx*	1.277900138	1.40E-05	0.000311593
Arhgef10	0.859945471	2.11E-05	0.000441189	*Pcnx3*	0.759528726	0.001244276	0.011106577
Arhgef11	1.045236838	0.00914045	0.048629868	*Pcnx4*	0.712494474	0.000302939	0.00371365
Arhgef16	3.874447208	0.00372608	0.024980693	*Pcyox1*	0.631652032	0.002107473	0.016340263
Arhgef18	0.675948729	0.002200036	0.016891662	*Pcyt1a*	0.69614645	0.000448247	0.005022652
Arhgef3	0.772409616	0.000321642	0.003875074	*Pcyt1b*	0.985920681	0.001145488	0.010445324
Arhgef38	5.925733629	0.002821671	0.02029606	*Pde3a*	1.284142959	0.000456453	0.005093471
Arhgef9	0.798839807	0.003337451	0.022991191	*Pde4dip*	1.246019783	0.001891179	0.015077586
Arid1b	1.021558105	5.45E-05	0.000951409	*Pdgfc*	0.554039915	0.001796004	0.014566934
Arid3a	0.910609704	0.000635786	0.00659147	*Pdha1*	0.514616128	0.00306769	0.021637514
Arih1	0.592966614	0.002460579	0.018304736	*Pdik1l*	0.476234709	0.006675817	0.038645174
Arih2	0.634138018	5.15E-06	0.000138844	*Pdpr*	1.068887775	7.41E-05	0.001211025
Arl5a	1.212246251	2.81E-07	1.18E-05	*Pds5b*	0.914786274	0.000974587	0.009248301
Arl5b	0.906944609	0.000157654	0.002225642	*Pdxdc1*	0.608929823	0.004465521	0.028662726
Armc6	0.63431471	0.000626687	0.006512512	*Pdzd8*	1.045595293	2.87E-07	1.20E-05
Armc9	0.526218445	0.005469951	0.033299878	*Pea15a*	0.890791901	2.08E-06	6.52E-05
Armcx1	0.710260709	3.39E-05	0.000643095	*Peg10*	0.653045501	0.001603337	0.01339871
Arsa	0.544059276	0.001544128	0.013042293	*Peg13*	1.338772276	2.04E-07	8.87E-06
Arsb	0.820496647	7.13E-05	0.001173602	*Peg3*	0.927740755	0.002538153	0.018719741
Arvcf	1.038637837	1.51E-05	0.000331681	*Peli3*	1.033829156	2.29E-05	0.000472639
Asb1	0.766633837	0.001547764	0.013067168	*Pelp1*	0.632171275	0.002469603	0.018357411
Asb13	0.531636771	0.004467087	0.028662726	*Pes1*	0.598008649	0.001809181	0.014633698
Asb7	0.837953383	0.000372674	0.004324524	*Pex26*	1.035698954	0.001548538	0.013067861
Ascc2	0.717437618	0.001737822	0.014220798	*Pex5l*	0.493017803	0.000421878	0.004798293
Asxl1	0.556318645	0.000905765	0.008722196	*Pfkl*	1.560658369	4.77E-13	6.44E-11
Atad2	0.9768496	0.007448983	0.041857353	*Pgap1*	1.069847884	6.80E-05	0.001130252

Atf2	0.871486427	7.78E-08	3.83E-06	*Pggt1b*	1.032142644	0.007058211	0.040223507
Atf6	1.244008161	0.000114402	0.001714948	*Phc1*	0.957932471	5.79E-07	2.18E-05
Atf7	0.944000497	0.000155881	0.00220518	*Phc3*	1.119342435	0.000120513	0.001792335
Atf7ip	0.801382304	0.000425915	0.004841295	*Phf3*	0.703944044	0.002641464	0.019308429
Atg13	0.543937143	0.002453352	0.018279744	*Phf6*	0.894736815	0.000344667	0.004083688
Atg14	0.564675889	0.001286703	0.011392293	*Phf8*	0.778010179	0.001350331	0.011789977
Atg2a	0.747665509	0.002718919	0.019752389	*Phka1*	0.887093	0.001189059	0.010732244
Atg2b	0.767664958	0.006865065	0.039419906	*Phlpp1*	0.645074686	0.004221327	0.027477874
Atg9a	0.661630293	0.000318478	0.003846763	*Phrf1*	1.030143064	4.08E-05	0.000749785
Atmin	0.715802708	0.001810821	0.014640697	*Pi4ka*	0.861052519	0.000594055	0.006245503
Atp11a	1.203626669	0.000301477	0.003702932	*Pianp*	2.263665999	0.003757315	0.025132528
Atp13a3	1.090883638	0.001866412	0.014955827	*Pias2*	0.575798451	0.002546894	0.018762638
Atp2a2	1.104994463	1.96E-05	0.000416	*Pigb*	0.651628098	0.002909834	0.02079569
Atp6v0a2	0.529625858	0.009257829	0.048951257	*Pigg*	0.733665381	0.007510093	0.042100619
Atp6v1g2	0.839443496	0.007389165	0.041613244	*Pigm*	0.570024255	0.008537916	0.046365124
Atp8a1	0.93837642	1.33E-05	0.000299647	*Pign*	0.676594599	0.005696427	0.034358504
Atrn	1.367321717	0.000115332	0.001724782	*Pik3c2a*	0.836746008	0.000809483	0.007969614
Atxn1l	1.312959469	3.73E-11	3.90E-09	*Pik3c3*	0.731722798	0.000846233	0.008245533
Atxn3	0.739630947	2.91E-05	0.000568774	*Pik3ca*	0.554228862	0.009341788	0.049284907
Avl9	1.25046935	5.14E-07	1.97E-05	*Pik3cb*	0.88504264	0.000179145	0.002455682
Axin1	0.759486114	0.005825104	0.035000617	*Pik3r1*	0.816700352	8.10E-06	0.000199009
Azi2	0.501328871	0.006887311	0.039523632	*Pik3r4*	0.990498356	6.49E-05	0.001090716
B3galt1	0.541532831	0.008646887	0.0467273	*Pip4k2a*	0.773730547	3.51E-05	0.000663037
B3galt5	1.061223773	0.000173253	0.002390513	*Pip4k2c*	0.738663085	8.09E-06	0.000199009
B3glct	0.597392797	0.004931681	0.030868839	*Pja1*	0.576508956	0.000364247	0.004244983
B4galnt1	1.338873825	0.000427745	0.00485177	*Pja2*	0.720507392	5.27E-05	0.000927408
Bag4	0.891360946	8.07E-05	0.001302622	*Pkn1*	0.71110443	0.00052556	0.005696481
Bag6	0.553550707	0.000217721	0.002854079	*Pkn2*	0.586353902	0.003320524	0.022891274
Bahd1	0.897501083	0.001607126	0.013412599	*Pknox1*	0.605201635	0.001400512	0.012065897
Bap1	0.56407311	0.003490168	0.023755907	*Plaa*	0.501713705	0.00439097	0.02828024
Bbs1	0.868567365	0.000619786	0.006451442	*Plagl1*	0.839650844	0.006112607	0.036217523
Bche	0.720404592	0.008502642	0.046242688	*Plagl2*	0.958203777	8.76E-05	0.001401121
Bcl2l11	0.912467535	4.05E-06	0.000113848	*Pld1*	0.663900921	0.001721716	0.01411618
Bcl2l13	0.653649097	0.000353174	0.004158908	*Plekha8*	0.783756045	0.000500021	0.005479359
Bcor	1.13585042	1.97E-07	8.63E-06	*Plekhg1*	0.732614345	0.000844213	0.008230095
Bdkrb2	2.571669997	4.95E-08	2.58E-06	*Plekhh1*	1.004642637	1.41E-07	6.41E-06
Bend3	1.03720615	0.006322541	0.0371266	*Plekhm1*	0.722570521	0.003652338	0.024604472
Bicc1	0.508208173	0.009482382	0.049887411	*Plekhm2*	1.076724862	1.07E-06	3.71E-05
Bicral	1.181743954	0.000199148	0.002676406	*Plekhm3*	0.815844117	0.000147485	0.002106813
Bmi1	0.588351416	0.002220006	0.016962315	*Plppr4*	4.185568662	1.32E-08	8.15E-07
Bmpr1a	0.878081215	8.75E-06	0.000213625	*Plppr5*	1.232135483	3.52E-08	1.92E-06
Borcs5	0.691571262	0.001048837	0.00981493	*Pls1*	0.710939277	0.006978218	0.03986378
Brap	0.625003515	6.35E-05	0.001076879	*Pls3*	1.065595915	6.79E-09	4.49E-07
Brd8	0.551512483	0.001656213	0.013695272	*Plxdc2*	0.59235146	0.006017299	0.035870388
Brinp3	0.874089211	0.001367383	0.01185944	*Plxnb1*	0.920549191	0.000292329	0.003604625
Brox	0.69868414	0.00018872	0.002560932	*Plxnb2*	1.033192947	0.001082611	0.010034435
Bsdc1	0.421320546	0.004078872	0.026743986	*Pnmal2*	0.755232964	0.000490082	0.005404913
Btbd11	1.512284504	0.006069478	0.036067695	*Pnpla7*	0.710794159	0.000339412	0.004036019
Btbd3	0.641793332	0.002968875	0.021129761	*Pnpla8*	0.667181128	9.10E-05	0.001433751
C1galt1	1.063043602	0.000825415	0.008092744	*Poc5*	0.660033391	0.000682208	0.006955652
C2cd3	1.194975049	2.29E-05	0.000472639	*Pogk*	0.965363693	5.45E-06	0.00014554
Cab39	0.84559362	1.54E-05	0.000338088	*Pola1*	0.684378907	0.00667699	0.038645174
Calcoco1	0.565076015	0.001154953	0.010526552	*Polg*	0.709859019	1.72E-05	0.000369307
Calu	0.530828775	0.003009267	0.021328902	*Polr2a*	0.898605663	0.000353561	0.004158908
Camsap1	0.820207611	0.003953269	0.026074198	*Polr2b*	0.687521919	0.003559964	0.024087022
Camsap2	1.320924766	0.000138652	0.00200378	*Polr2m*	0.498206641	0.008946399	0.047894016
Cand1	1.026981194	9.40E-06	0.000226771	*Polrmt*	0.648763995	0.002880322	0.020623761
Caprin1	0.870645032	0.00060569	0.006329113	*Pou6f1*	1.043980616	0.000266426	0.003346343
Carhsp1	0.471582751	0.009242787	0.04891807	*Pphln1*	0.667475766	1.59E-05	0.000345477
Casc3	0.783107932	0.000130533	0.001906858	*Ppm1f*	0.733746181	0.000491494	0.005414172
Caskin2	1.000149553	4.68E-07	1.82E-05	*Ppm1h*	0.46137449	0.0050769	0.03150645
Castor2	0.743802644	9.08E-05	0.001432656	*Ppm1k*	0.648257089	0.001296434	0.011451557
Cbfb	0.649808904	0.004509505	0.028904102	*Ppm1l*	0.750967161	0.001071285	0.009960897
Cblb	0.573165389	0.005002179	0.031189386	*Ppp1r12b*	0.906149594	0.005424016	0.033108009
Cbx4	0.548513139	0.000112758	0.001694327	*Ppp1r13b*	1.115709432	9.16E-09	5.85E-07
Cbx5	1.007731284	5.39E-05	0.000944762	*Ppp2r2c*	0.869534738	0.000389512	0.004478674
Ccar2	0.660257473	0.000180642	0.002472616	*Ppp2r3a*	0.669528589	0.000672427	0.006878186
Ccdc186	0.806002579	0.001276343	0.011321783	*Ppp2r5c*	0.705797753	0.000649916	0.006705981
Ccdc47	0.903568098	4.37E-06	0.000120992	*Ppp4r3a*	0.665189731	0.002049531	0.015992143
Ccdc82	0.869517656	2.24E-05	0.000464034	*Ppp4r3b*	0.609789342	0.000351737	0.004147773

Ccdc93	1.22056021	9.97E-05	0.001536168	*Ppp6r3*	0.831398514	0.000323804	0.003896161
Ccdc97	0.611001845	0.001024054	0.009630696	*Pprc1*	0.67637708	0.00541592	0.033099624
Ccng1	0.748555559	0.000207346	0.002748574	*Prag1*	1.579745231	1.18E-14	1.92E-12
Cd164	0.567397158	0.002120052	0.01639073	*Prdm10*	0.727263185	0.000913741	0.008785578
Cd200	0.651043844	0.000819123	0.008056128	*Prdm2*	0.934194374	6.70E-05	0.00111606
Cd2ap	1.199019569	9.71E-05	0.001510732	*Prepl*	0.788079096	4.25E-05	0.00077642
Cd2bp2	0.417423847	0.005223117	0.032191908	*Prex1*	0.896070124	3.74E-05	0.000698695
Cd99l2	0.855647662	5.33E-05	0.000937356	*Prickle2*	0.844953873	5.44E-05	0.000951002
Cdc23	0.585485006	0.001310012	0.011544597	*Prkacb*	1.032967479	9.85E-07	3.46E-05
Cdc25a	1.481301005	1.51E-05	0.000332828	*Prkca*	0.953284014	0.004162187	0.027189952
Cdc27	0.68028963	0.002861847	0.020522573	*Prkci*	1.044478484	9.47E-07	3.34E-05
Cdc42bpa	0.68568634	0.002747357	0.019913068	*Prkx*	0.533353137	0.001610307	0.013427277
Cdc73	0.446146237	0.007252192	0.041019803	*Prlr*	2.285977124	1.66E-07	7.44E-06
Cdh2	0.84641743	0.002085332	0.016195163	*Prob1*	0.784385012	8.39E-07	3.00E-05
Cdh3	7.10639665	6.53E-05	0.001094433	*Prox1*	0.782136899	0.000681075	0.00695142
Cdk13	0.462525007	0.006568363	0.038227144	*Prox2*	0.87986809	1.45E-05	0.000322212
Cdk16	0.476186079	0.008742191	0.047125088	*Prpf18*	0.597999725	0.000648385	0.006693836
Cdkn2aip	0.539316674	0.007181844	0.040756781	*Prpf19*	0.382069793	0.007194887	0.040767723
Cdr2l	0.714827352	5.47E-05	0.000953892	*Prpf6*	0.585667053	0.005368766	0.032864567
Cds2	0.865174511	0.000160848	0.002252231	*Prpf8*	0.527222795	0.008641722	0.046726109
Cecr2	0.664967601	0.000173658	0.002394356	*Prps2*	0.719431511	0.0011183	0.010286729
Celf1	0.642704866	0.000277549	0.003461903	*Prr14l*	0.595433995	0.000171089	0.002367563
Cep104	0.866182363	1.58E-05	0.000343514	*Prrc1*	0.533765104	0.00196895	0.015572832
Cep120	0.920553211	0.000761582	0.007606143	*Prss56*	5.097401196	3.87E-09	2.73E-07
Cep170	1.069412688	2.52E-07	1.07E-05	*Prxl2a*	0.533247177	0.000146828	0.002101056
Cep170b	1.104526866	9.07E-08	4.34E-06	*Psd3*	1.05571812	5.88E-05	0.001012729
Cep68	0.684303483	0.003819056	0.025428534	*Psen2*	0.638597886	0.002109806	0.016351647
Cep85	0.642863321	0.00182977	0.014752384	*Psip1*	0.509637241	0.004528582	0.028978942
Cerk	0.656402633	0.002119311	0.01639073	*Psmd2*	0.475891424	0.006301587	0.037039459
Cfap46	1.257010902	0.004000974	0.026342882	*Ptar1*	0.880729196	0.000342575	0.004065092
Cflar	1.150178674	7.79E-06	0.000192863	*Ptcd1*	0.833547197	0.004330688	0.028006496
Chaf1a	0.725605802	0.002458913	0.018299539	*Ptch1*	1.190570075	9.71E-09	6.16E-07
Champ1	0.784796722	0.000219454	0.00287281	*Ptpdc1*	0.594542424	0.006845089	0.039317141
Chd1	0.75213807	1.80E-05	0.000384662	*Ptpn21*	0.677110374	0.009489099	0.049908859
Chd4	0.682018774	0.000107183	0.001631307	*Ptpra*	0.558581923	0.007282807	0.041156027
Chdh	1.664528639	3.75E-20	1.18E-17	*Ptpre*	0.676870929	0.000449283	0.005031279
Chil1	1.489209785	7.89E-06	0.000194529	*Ptprf*	0.796883137	7.28E-06	0.000182944
Chml	1.067462105	0.002717888	0.019752389	*Ptprg*	1.05610829	0.004090867	0.026799812
Chmp4b	1.130113915	2.76E-06	8.21E-05	*Ptprk*	0.839156393	0.000376007	0.004355181
Chpt1	0.704954297	0.000495382	0.005444305	*Ptprm*	1.409213703	5.29E-06	0.000142051
Chst14	0.596654615	0.002264555	0.017191517	*Ptprs*	0.494928976	0.006535042	0.038091861
Chst3	1.598878388	5.26E-11	5.26E-09	*Pum1*	0.608029771	0.004508614	0.028904102
Ckap5	0.979210843	3.75E-05	0.000699635	*Purg*	1.200623902	6.84E-06	0.000174488
Clasp2	0.740072506	0.000355284	0.004168802	*Pwp2*	0.502518329	0.008575103	0.0464989
Clcn5	1.094122512	2.03E-07	8.87E-06	*Pxdn*	3.610724435	1.77E-15	3.19E-13
Clcn6	0.670030558	0.003879982	0.025733109	*Pxmp4*	0.763856591	0.003928515	0.025924792
Clcn7	0.613963141	0.008197776	0.045105333	*Qsox2*	0.467036137	0.009192633	0.048825048
Cldn5	8.122916383	4.90E-08	2.57E-06	*Rab10os*	0.614096909	0.00149482	0.012699586
Clic5	2.894207917	6.80E-17	1.43E-14	*Rab11fip4*	1.043307174	0.000163839	0.002280592
Clint1	0.517652145	0.007155875	0.040657501	*Rab22a*	0.60898322	0.002176808	0.016763474
Clip2	0.631715987	0.002114586	0.016372461	*Rab23*	0.785289945	0.000433194	0.004900456
Clock	0.87544434	7.70E-06	0.00019074	*Rab3gap1*	0.700690046	0.002014813	0.015808343
Clstn1	0.734036214	0.00418338	0.02731552	*Rab5a*	0.629241234	6.52E-05	0.001094423
Cltc	0.88033458	0.00018306	0.002502081	*Rab8b*	0.63074422	0.004738684	0.029998441
Cnot1	1.14378353	6.45E-06	0.00016806	*Rabgap1*	0.725906535	1.75E-05	0.000375284
Cnst	0.753325595	0.002573748	0.018923201	*Rabgap1l*	0.629987539	0.000811561	0.007985912
Cntn2	0.970390559	2.06E-05	0.000430941	*Rabgef1*	0.483623641	0.002227472	0.016991889
Coa5	0.747419221	0.006043689	0.035959666	*Rad21*	0.591790482	0.000172096	0.002376289
Cobl	0.826696657	6.02E-05	0.001031041	*Rad51d*	0.433001815	0.004736922	0.029997331
Cobll1	0.880681009	0.00012467	0.001839687	*Ralgds*	0.826781603	0.000881905	0.00852719
Cog3	0.625444947	0.001998322	0.015706563	*Ranbp2*	1.190328902	3.48E-07	1.42E-05
Col6a3	1.228076149	0.000455318	0.005085695	*Rapgef2*	1.097593122	3.14E-07	1.30E-05
Col9a2	0.900782887	0.005954808	0.035587648	*Rapgef3*	0.489039586	0.007699521	0.04296457
Copb1	0.51671084	0.008411839	0.045876799	*Rapgef5*	0.793850599	0.005591372	0.033854486
Copg1	0.767705146	0.001191145	0.010737349	*Rasa3*	0.727708278	0.000557826	0.005947315
Cops2	0.422418993	0.009023425	0.048197177	*Rasgrp3*	0.45927542	0.003220544	0.022417851
Coq8a	0.714131445	7.23E-05	0.001186943	*Rassf2*	0.801012865	0.000356543	0.004178389
Coro1c	0.626239561	1.71E-05	0.000369286	*Rassf3*	0.936671019	0.003185765	0.022257101
Coro2b	0.774509948	0.006094146	0.03613477	*Rassf4*	1.273509229	1.44E-08	8.76E-07
Cox15	0.459468123	0.004121888	0.02696057	*Rb1*	0.803020272	0.002069124	0.016115634

Cpd	1.052385664	0.000244328	0.003126966	*Rb1cc1*	0.654106912	0.002368763	0.017826308
Cpeb2	0.87029724	0.000457171	0.005098472	*Rbak*	0.604244296	0.007959483	0.044109675
Cpeb3	0.733078453	0.000656038	0.0067544	*Rbbp5*	0.573596751	0.005240785	0.032237732
Cpeb4	1.025652363	0.00018794	0.002555573	*Rbl2*	1.309359088	6.30E-09	4.22E-07
Cpm	0.551487323	0.003240967	0.022518465	*Rbm15b*	1.014380354	1.16E-05	0.000267224
Cpne3	0.827390328	0.004223102	0.027479965	*Rbm41*	1.031640316	5.89E-06	0.000155412
Cpped1	0.650607105	0.00016077	0.002252231	*Rbsn*	0.83878672	0.001067454	0.009954658
Cpsf6	0.867680997	2.32E-07	9.96E-06	*Rc3h2*	0.648179539	0.000731079	0.007370445
Creb1	0.823123702	0.000328753	0.00394156	*Rcan3*	0.751011754	0.000879982	0.008517303
Creb3l2	0.743434755	0.001260411	0.011196215	*Rcbtb1*	0.541598113	0.003733851	0.025010977
Crebl2	1.354500373	4.87E-10	4.11E-08	*Rcc2*	1.112543528	0.000354467	0.004163293
Crebrf	0.667521247	0.003147554	0.022085456	*Rdx*	0.561456945	0.00077746	0.007712992
Crk	0.782169327	8.99E-05	0.001423473	*Recql*	0.615037394	0.004786845	0.030242556
Cry1	0.964433332	0.003864788	0.025669741	*Reps2*	1.047115838	0.003037776	0.021488736
Crybg3	1.034803325	0.002298203	0.017384129	*Rev1*	0.795631298	0.001329936	0.011676626
Csde1	0.680316749	7.20E-05	0.001183115	*Rffl*	0.44631653	0.006454157	0.037711404
Csnk1d	0.59091304	0.000339144	0.004036019	*Rftn1*	0.700044874	3.93E-07	1.57E-05
Cstf2	1.027488068	1.04E-07	4.88E-06	*Rftn2*	0.63315835	0.000998195	0.009415613
Ctbs	0.5599284	0.005421292	0.033108009	*Rfwd3*	0.602200975	0.008166539	0.045045837
Ctc1	0.501262873	0.009067048	0.048334575	*Rfx7*	1.066877684	0.000466544	0.00517856
Ctdp1	0.709579161	0.000147776	0.002107406	*Rgs3*	0.576943836	0.000538534	0.00579392
Cth	2.53994264	1.81E-06	5.85E-05	*Rhbdd1*	0.961682175	1.34E-05	0.000300879
Ctnna1	0.911312648	1.16E-05	0.000267224	*Rhobtb3*	0.791029221	0.005512284	0.033470815
Ctnnb1	0.719977357	0.000194955	0.002628549	*Rhoq*	0.572231158	0.000602085	0.006301888
Ctnnd1	0.508927215	0.007009429	0.04000581	*Rhpn2*	0.947324123	2.82E-05	0.000554167
Cttn	0.540294543	0.005356119	0.032819034	*Ric1*	0.605030806	0.009463057	0.049799603
Cttnbp2nl	1.138445286	1.80E-07	8.00E-06	*Ric3*	0.494243332	0.009052938	0.048274193
Cul3	0.578144358	0.007182867	0.040756781	*Ric8b*	0.559993234	0.005281855	0.032427057
Cul4a	0.672788809	0.000255451	0.003241825	*Rimkla*	0.562682328	0.006451356	0.037711404
Cul4b	0.958983324	2.45E-06	7.43E-05	*Rin2*	0.671505944	2.80E-05	0.000550413
Cul5	0.497022117	0.005828282	0.035008582	*Rlim*	1.042709452	5.90E-05	0.001015686
Cul7	0.586780889	0.006799984	0.039141318	*Rmnd5a*	1.331877679	3.01E-08	1.67E-06
Cxcr4	2.757848797	6.68E-10	5.44E-08	*Rnase1*	2.54877994	5.02E-05	0.000894366
Cyfip1	0.616571637	0.000147565	0.002106813	*Rnf125*	0.825824515	0.007133326	0.040590384
Cyld	0.596584129	0.004516602	0.028921859	*Rnf14*	0.490025101	0.001289174	0.01140883
Cyp26b1	1.375622617	0.003860321	0.025649083	*Rnf141*	0.542182812	0.001472276	0.012557286
Cyp2d22	1.109276659	0.008479537	0.046166098	*Rnf144a*	1.31398896	9.71E-08	4.58E-06
Dcaf1	0.982789403	1.23E-07	5.68E-06	*Rnf145*	0.564678435	0.009036013	0.04823715
Dcaf5	0.786267968	6.40E-05	0.001080395	*Rnf168*	1.002043012	1.42E-05	0.000316242
Dcaf7	0.760905445	5.69E-05	0.000984008	*Rnf2*	0.527452456	0.005847728	0.035103079
Dcbld1	0.899483229	0.002177132	0.016763474	*Rnf217*	2.086845896	0.005574478	0.033773831
Dcp1a	0.522814891	0.003227892	0.022449166	*Rnf24*	1.273717833	4.22E-07	1.67E-05
Dctn1	0.789600416	0.001072535	0.009967619	*Rnf38*	0.528282728	0.002188748	0.016832345
Dcun1d4	0.886474531	1.27E-06	4.26E-05	*Rnf43*	0.667997526	0.000192115	0.002594315
Ddb1	0.713062666	0.000152615	0.002167447	*Rnf44*	0.891377681	0.001069888	0.0099577
Ddx19b	0.672600826	0.003385627	0.023229948	*Rngtt*	0.666595881	0.000861515	0.008364261
Ddx21	0.511243168	0.006275631	0.036978879	*Rp2*	0.818429093	7.13E-05	0.001173732
Ddx42	0.55573724	0.001334267	0.011687512	*Rprd1a*	0.521607218	0.002063823	0.016080973
Ddx59	0.525853969	0.001535052	0.012981041	*Rprd1b*	0.504045183	0.001701352	0.014007253
Dennd2a	0.81559269	0.000183514	0.002506482	*Rps6ka1*	0.942997969	0.000109512	0.001660074
Dennd4a	0.967524309	6.60E-05	0.001101518	*Rps6ka5*	0.682756227	0.00165394	0.013682462
Dennd5a	0.624836017	0.002430197	0.018161438	*Rps6kc1*	0.681519448	0.000764093	0.007621975
Dgcr8	0.881113005	0.00014036	0.002020732	*Rrm2b*	1.064152908	2.83E-06	8.40E-05
Dgkd	0.810629285	0.000201698	0.00270086	*Rrn3*	0.914782799	0.000302072	0.003705636
Dgke	0.913268932	0.000184118	0.002512914	*Rrp12*	0.72913411	0.000741024	0.007441866
Dhx30	0.520974251	0.000444346	0.00498189	*Rs1*	1.226926409	0.000857137	0.008338885
Dhx37	0.54455333	0.001985935	0.015634564	*Rtl5*	1.069610351	0.000995756	0.009403807
Dhx38	0.827903754	0.00693225	0.039683879	*Rtl6*	0.572735198	0.00250482	0.018517248
Dhx40	0.459688207	0.002792383	0.020142432	*Rtn4*	0.577254986	0.001962765	0.015549933
Dhx8	0.56267678	0.00369167	0.02481637	*Rubcn*	0.882483128	0.001518519	0.01286047
Dicer1	0.617835928	0.003466483	0.023647392	*Rufy3*	0.483950373	0.007729921	0.043095715
Dip2a	1.075358455	0.000150717	0.002143477	*Rufy4*	9.144087157	9.78E-14	1.44E-11
Dip2c	0.862406817	0.000216323	0.002841663	*Rusc2*	0.658611048	0.000639867	0.006623993
Dipk2a	0.898811019	0.000207836	0.002752018	*Rwdd3*	1.409306851	2.15E-07	9.29E-06
Diras1	0.687195643	0.000147829	0.002107406	*Rybp*	0.745947349	5.09E-06	0.000137902
Disp3	2.24582536	3.99E-06	0.000112106	*Sall1*	0.601756828	0.00369696	0.024843096
Dixdc1	1.156384299	1.55E-06	5.06E-05	*Samd8*	1.398480737	2.48E-12	3.08E-10
Dlg1	0.577026842	0.005978255	0.035701792	*Sart3*	0.45317464	0.006455806	0.037711404
Dlg2	1.367507037	8.97E-08	4.32E-06	*Sbf2*	0.771401827	6.15E-05	0.001049066
Dlk2	3.520534307	1.87E-40	2.08E-37	*Sbk1*	0.834778152	2.63E-05	0.000523193

Dll1	1.362063762	3.48E-05	0.000657956	*Scaf8*	0.547768713	0.000775926	0.007708137
Dmxl1	0.759517876	0.004293964	0.027826124	*Scamp1*	0.575513659	0.008334756	0.04558793
Dnaaf5	0.537737968	0.006515814	0.038026684	*Scd2*	0.79637038	0.002422753	0.018137541
Dnah14	1.262440936	0.001890508	0.015077586	*Scn3b*	1.098729557	0.000782016	0.007747617
Dnah8	0.999508357	0.008590376	0.046555011	*Scrg1*	3.015881015	1.54E-09	1.17E-07
Dnaja4	0.480549165	0.007496903	0.042039143	*Scyl2*	0.753657642	0.000158484	0.002232353
Dnajb14	1.055699042	9.28E-06	0.000224611	*Scyl3*	0.658652596	2.49E-05	0.000503267
Dnajc11	0.405539171	0.008405294	0.045876799	*Sec14l1*	0.517723148	0.002074061	0.016147435
Dnajc16	1.118897401	1.75E-07	7.79E-06	*Sec16a*	0.688293842	0.00456259	0.029147225
Dnajc18	0.594415711	0.003267698	0.022626517	*Sec23a*	0.557099932	0.001466698	0.012522578
Dnajc27	0.603543596	0.001464389	0.012508514	*Sec24b*	0.65697718	0.000765827	0.007627197
Dnajc28	0.812178181	1.54E-05	0.000338088	*Sec24c*	0.609712438	0.008177868	0.04506599
Dnajc3	0.637404495	0.003799523	0.025334176	*Sec63*	0.790915746	0.000205758	0.002740972
Dnajc5	0.643224855	0.000198008	0.002664023	*Secisbp2l*	0.879030192	1.06E-05	0.000250879
Dnajc6	0.873983139	4.10E-07	1.64E-05	*Sel1l*	0.84780499	2.62E-05	0.000521473
Dnm2	0.789914549	0.001293743	0.011443905	*Sel1l3*	1.004006499	3.53E-05	0.000665669
Dnmt1	0.891101386	0.001875761	0.014998949	*Selenoi*	0.754812254	0.000163348	0.002277115
Dntt	1.466724135	0.005374971	0.032884694	*Selenon*	1.053939032	3.67E-05	0.000689324
Dock1	1.108871675	2.12E-07	9.20E-06	*Sema3d*	1.003170446	7.41E-06	0.000184843
Dock10	0.877667618	0.00046221	0.005145553	*Sema3e*	0.995591958	0.000382599	0.004418005
Dock5	1.307758036	0.000125557	0.001848447	*Sema6d*	1.725339456	1.25E-16	2.61E-14
Dop1b	0.797090369	0.006816066	0.039210011	*Senp1*	0.582488306	0.000978065	0.009271996
Dpp8	0.951882099	6.90E-05	0.001143947	*Sephs1*	0.74122001	0.001505916	0.012788111
Dpy19l1	0.846317655	0.00267793	0.019522142	*Sepsecs*	0.683578604	0.000236937	0.003055135
Dpy19l4	0.699769434	0.001443753	0.012365776	*Serinc1*	0.533149066	0.009409262	0.049585523
Dpysl5	0.957335716	0.000166518	0.002311076	*Sestd1*	0.687489644	0.00193196	0.015350924
Dr1	0.464567313	0.004885877	0.030642912	*Setd7*	0.741638674	0.001360328	0.011822659
Drosha	0.805705301	0.004985673	0.031144805	*Setx*	0.849255294	9.75E-05	0.001515112
Dscaml1	0.829003579	0.001914545	0.015231749	*Sf1*	0.660080072	0.003181531	0.022246344
Dtx4	1.258114213	4.08E-09	2.86E-07	*Sf3a1*	0.564708797	0.003196124	0.022302078
Dync1h1	0.83701422	0.001712887	0.014059355	*Sf3b2*	0.393285244	0.003363408	0.023127864
Dync1i1	1.359092294	1.34E-05	0.000300619	*Sf3b3*	0.629701835	0.001134801	0.010377913
Dync1li2	0.946311948	0.000679785	0.006942185	*Sfrp1*	0.638171649	0.001411388	0.012138064
Dyrk1a	0.659810554	0.001397539	0.012046366	*Sft2d2*	0.926915486	2.61E-05	0.000521338
Dzank1	1.024347913	0.004435591	0.028489968	*Sgcb*	0.752962823	0.000398712	0.004573338
E2f2	0.912757089	0.008245242	0.045271331	*Sgms2*	1.067579547	0.000969699	0.009215802
Eaf1	1.077320969	0.000281687	0.003499316	*Sgpp1*	0.835871209	6.38E-05	0.00107914
Edem3	1.444191178	1.76E-10	1.65E-08	*Sgsm1*	0.938056336	0.000135791	0.00196845
Edil3	1.078994569	2.29E-05	0.000472639	*Sgtb*	0.636626034	0.006590049	0.038341548
Edrf1	0.783749256	0.007219279	0.040879257	*Sh2b2*	1.734060229	0.000365442	0.004253664
Efs	0.918878524	0.003792506	0.025300797	*Sh3bgrl2*	0.957947556	6.42E-05	0.001082118
Eftud2	0.505209344	0.002782385	0.020082254	*Sh3bp5l*	0.811129757	2.91E-07	1.22E-05
Eif2ak1	0.693897153	0.000249837	0.003188839	*Sh3kbp1*	0.76822793	0.003307197	0.022832704
Eif2ak3	1.069699744	7.48E-07	2.71E-05	*Sh3rf1*	0.968722887	5.78E-05	0.000996912
Eif4b	0.478173826	0.006948833	0.03975599	*Sh3tc2*	0.571453047	0.00158315	0.013294661
Eif4g1	0.835954281	0.00165256	0.01368182	*Shisa2*	3.621137082	0.000227905	0.002960881
Eif4g2	0.946511265	0.000129858	0.001898454	*Shoc2*	0.840217112	5.13E-05	0.000908028
Eif4g3	0.960850044	0.00314967	0.022092101	*Shprh*	0.813087649	0.000867776	0.008420726
Eif5a2	1.08221465	6.02E-08	3.06E-06	*Shroom2*	0.9027267	8.93E-06	0.000216893
Elac1	0.849734658	0.001167622	0.010611323	*Simc1*	0.914230568	0.001332593	0.011683675
Elf2	0.44432286	0.00470117	0.029820878	*Sipa1l2*	1.200317695	3.30E-06	9.59E-05
Elk4	0.821261324	1.37E-05	0.000305252	*Sipa1l3*	1.005447365	4.49E-05	0.000812301
Ell2	0.567986136	0.005927219	0.035478853	*Slain2*	0.851694894	0.004229963	0.027508679
Elmo1	0.743971768	2.19E-06	6.79E-05	***Slc11a2***	**0.631278766**	**4.23E-06**	**0.000118005**
Elmod2	1.834642597	1.73E-22	6.55E-20	***Slc12a2***	**0.787369063**	**0.002936728**	**0.020948288**
Elmsan1	0.586270815	0.009040901	0.04824186	***Slc16a2***	**1.024234407**	**0.00502688**	**0.031309098**
Elp1	0.704575802	0.004883592	0.030642912	***Slc17a5***	**0.503969957**	**0.00693413**	**0.039683879**
Emc8	0.468304195	0.004398304	0.028309809	***Slc19a1***	**0.684153076**	**0.001068604**	**0.0099577**
Emilin2	0.888709695	0.00071918	0.007265985	***Slc20a2***	**0.589850099**	**0.000363164**	**0.00423506**
Eml1	0.673908961	0.001508489	0.012804207	***Slc22a23***	**1.018421967**	**0.002634303**	**0.019263535**
Endod1	0.805105345	4.27E-05	0.000777211	***Slc25a23***	**0.550772549**	**0.000337004**	**0.004019168**
Eno1b	3.54279075	2.37E-29	1.49E-26	***Slc25a44***	**0.569948599**	**0.001770193**	**0.014410832**
Eno2	2.068003373	1.55E-17	3.53E-15	***Slc27a1***	**0.781293763**	**0.000317264**	**0.003837012**
Enpp1	0.704199953	0.005471588	0.033299878	***Slc30a1***	**0.893173184**	**0.000376628**	**0.00435971**
Enpp2	0.72873454	0.00125462	0.011153207	***Slc30a2***	**4.038609295**	**1.30E-13**	**1.90E-11**
Entpd5	0.925266179	1.06E-05	0.000250819	***Slc30a4***	**1.135171833**	**0.00095394**	**0.009105828**
Eny2	0.430532589	0.003631622	0.024499837	***Slc30a7***	**1.216562978**	**5.63E-07**	**2.13E-05**
Epas1	1.164138548	2.33E-05	0.00047653	***Slc33a1***	**0.55546545**	**0.00776121**	**0.043251813**
Epb41	1.373068681	0.000518934	0.005647327	***Slc35a3***	**0.514524227**	**0.00813025**	**0.044898074**
Epg5	0.794277488	0.000396578	0.004551623	***Slc35c1***	**0.691593057**	**0.003452842**	**0.023571351**

Epha5	1.500003654	0.001835602	0.014784141	***Slc35e1***	**0.917979686**	**1.07E-05**	**0.000251136**
Epm2aip1	1.009315522	1.18E-05	0.000271512	***Slc35e3***	**0.566307449**	**0.004212743**	**0.027431443**
Eprs	0.401394794	0.007072484	0.040292697	***Slc36a1***	**1.767894304**	**3.31E-06**	**9.60E-05**
Eps15	1.16881531	3.55E-07	1.44E-05	***Slc36a4***	**0.974969493**	**6.31E-06**	**0.000164912**
Erbb3	1.045626589	3.78E-07	1.52E-05	***Slc38a10***	**0.634241001**	**4.57E-05**	**0.000824144**
Erbb4	0.991635487	3.24E-07	1.34E-05	***Slc38a2***	**1.053912347**	**2.71E-05**	**0.000537345**
Erbin	1.038952445	6.98E-07	2.56E-05	***Slc39a10***	**0.622072676**	**0.002560887**	**0.018835975**
Erc1	0.617022943	0.008857642	0.047499574	***Slc39a14***	**0.541933563**	**0.001089506**	**0.010076721**
Erlec1	1.255402354	3.21E-09	2.29E-07	***Slc39a3***	**0.439244784**	**0.007172694**	**0.040740814**
Ermp1	0.90909393	0.000282051	0.003499316	***Slc39a6***	**0.737393025**	**0.000264855**	**0.003331043**
Ern1	1.121033679	7.68E-08	3.79E-06	***Slc39a9***	**0.901133818**	**6.13E-06**	**0.000160655**
Ero1l	0.876522496	6.79E-06	0.000173386	***Slc44a1***	**0.670698223**	**0.002195907**	**0.016873674**
Esyt2	0.932633376	8.97E-05	0.001423031	***Slc44a2***	**0.755973274**	**5.44E-06**	**0.000145405**
Etnk1	1.204577297	7.12E-06	0.000180089	***Slc4a2***	**0.46902593**	**0.002750974**	**0.019917538**
Etv5	1.441569974	5.98E-06	0.000157549	***Slc5a11***	**0.902508946**	**0.000791273**	**0.00781884**
Exoc2	0.659251247	0.000779024	0.007722016	***Slc5a3***	**1.616318766**	**8.24E-10**	**6.60E-08**
Exoc6	0.644650481	0.002258684	0.017173587	***Slc6a1***	**1.19684599**	**2.58E-05**	**0.000516005**
Exoc6b	0.910406918	0.000242958	0.003113646	***Slc6a6***	**0.628480128**	**0.000503617**	**0.005502813**
Extl2	0.501909229	0.006042444	0.035959666	***Slc8a1***	**0.624294647**	**0.001173266**	**0.010647265**
Extl3	0.735428031	0.000279258	0.003477478	***Slc9a6***	**0.812451729**	**0.000206922**	**0.002748105**
Ezh1	0.803666628	1.07E-05	0.000251274	***Slc9a8***	**0.801236964**	**9.92E-05**	**0.001533925**
F5	5.016017848	8.95E-05	0.001423031	*Slco1a4*	3.388648171	5.41E-06	0.000144801
Fa2h	0.549843879	0.00523677	0.032228714	*Slf2*	1.228557213	1.36E-06	4.53E-05
Fads1	0.829684054	0.001343642	0.011764177	*Slmap*	0.644008001	0.000715656	0.007241989
Fah	0.428783422	0.001620023	0.013492932	*Slx4*	1.24751922	0.003260092	0.022598284
Fam117b	1.059544514	1.16E-08	7.25E-07	*Smad2*	0.609076192	0.005006136	0.031190172
Fam120b	0.483549897	0.001979808	0.015619496	*Smad3*	1.446119765	8.41E-09	5.44E-07
Fam120c	1.241427319	1.17E-05	0.00026942	*Smad5*	1.007674233	9.33E-06	0.000225623
Fam122b	0.973652308	6.43E-05	0.00108233	*Smad9*	1.123397312	0.002164118	0.016676851
Fam126a	0.785277123	0.000436521	0.004927968	*Smarca2*	0.795118525	0.001563987	0.013174706
Fam129b	0.733033483	0.000403016	0.004616275	*Smarca5*	0.571062702	0.008904626	0.047710925
Fam131b	0.617574993	0.00644664	0.037704469	*Smarcal1*	0.601809877	0.000230021	0.002986327
Fam160b1	0.793226026	4.15E-06	0.00011634	*Smarcc1*	0.593801167	0.002395608	0.017977045
Fam168a	0.644707734	0.002725844	0.019787491	*Smarcc2*	0.956262848	0.003558589	0.024087022
Fam168b	0.968492179	0.008228579	0.045203724	*Smc1a*	0.6097738	0.00025198	0.003211855
Fam171a1	0.741049452	0.001012056	0.009532089	*Smcr8*	1.178571451	2.57E-05	0.000513914
Fam171b	0.746290432	9.04E-05	0.001428361	*Smg7*	0.750785434	0.000249542	0.003187219
Fam189a2	0.536855416	0.007576438	0.042384552	*Smim10l1*	0.717386282	7.53E-06	0.000187401
Fam20b	0.791086145	4.28E-06	0.000119237	*Smim13*	0.933785568	5.63E-07	2.13E-05
Fam210b	0.770997438	0.001333576	0.011686873	*Smoc1*	1.301923794	2.31E-11	2.54E-09
Fam219b	0.539085664	0.004343868	0.028072525	*Smug1*	0.542296891	0.001312204	0.011558526
Fam32a	0.511710553	0.000188326	0.002557413	*Smurf1*	0.589930275	0.004633504	0.029470768
Fam45a	0.743503574	0.00046576	0.005174879	*Smyd3*	0.62767989	0.001075906	0.009987248
Fam53b	0.632155836	0.000128698	0.001882962	*Snap29*	0.544969989	0.006521341	0.03803056
Fam53c	0.76860034	3.19E-05	0.000612503	*Snd1*	0.665859761	1.80E-05	0.000384804
Fam8a1	0.901401586	9.08E-05	0.001432656	*Snn*	1.012860668	5.43E-07	2.07E-05
Fam91a1	1.173750986	3.43E-06	9.82E-05	*Snrnp200*	0.739140565	9.78E-05	0.001516573
Far1	0.480765208	0.008143672	0.044959061	*Sntb1*	3.577255899	0.001885586	0.01504569
Farp2	0.796327211	0.000209959	0.002773483	*Snx13*	0.854848039	3.02E-05	0.000585735
Fat1	0.879504731	0.001047636	0.009808547	*Snx18*	0.585724462	0.005901625	0.035359245
Faxc	1.763936744	0.001710644	0.014049574	*Snx19*	0.894651315	1.53E-06	5.01E-05
Fbln2	3.573122395	0.005066414	0.031480063	*Snx25*	1.502080168	0.00736733	0.041534339
Fbxl3	0.541822119	0.002495204	0.018457389	*Snx30*	0.531535017	0.000604799	0.006323292
Fbxo10	0.978210803	0.000453809	0.005075953	*Socs4*	1.195282793	2.08E-06	6.52E-05
Fbxo21	0.685664199	1.93E-05	0.000410063	*Socs5*	0.613880754	0.002319794	0.017512403
Fbxo32	0.478326872	0.002993612	0.021249811	*Socs6*	0.591528664	0.002259179	0.017173587
Fbxo34	0.645459304	0.000348793	0.004120772	*Soga1*	0.712660674	9.75E-05	0.001515112
Fbxo38	0.866590102	1.17E-06	3.99E-05	*Sorl1*	0.582462942	0.008330268	0.04558793
Fbxo45	1.15143936	2.78E-05	0.000547229	*Sort1*	1.248696844	2.02E-06	6.38E-05
Fbxw11	0.943298908	1.96E-05	0.00041533	*Sos1*	0.936393756	0.001017629	0.009579805
Fcho1	7.142669447	1.38E-93	6.52E-90	*Sos2*	0.843935432	0.001346657	0.011774218
Fcho2	0.790522498	0.000157996	0.002228811	*Sowahc*	0.905291469	0.000304817	0.00373425
Fem1c	0.702450188	1.07E-05	0.000251274	*Sox10*	0.548649904	0.003222391	0.022419159
Fer1l5	1.336359719	0.001753725	0.014313759	*Sox12*	0.77095447	0.002242531	0.017072314
Fgf1	1.378274167	2.81E-10	2.48E-08	*Sox13*	1.243153035	0.002515685	0.018575777
Fgf7	1.3568992	0.00032973	0.003947661	*Sp2*	0.728439706	6.26E-05	0.001065344
Fgfr2	0.693509167	0.000121399	0.001801898	*Sp3*	0.625110243	0.001180379	0.010692419
Fip1l1	0.801864622	0.000990055	0.009359735	*Sp4*	0.719454239	0.002306987	0.017436617
Fitm2	0.562890826	0.008791419	0.047292028	*Spag9*	1.231636623	1.58E-05	0.000344367
Flnb	1.401355197	5.88E-06	0.000155186	*Spast*	0.666892326	0.00033191	0.003965925

Flt1	6.974431481	2.50E-06	7.52E-05	*Spata2*	0.664743402	4.95E-06	0.000134675
Flywch1	0.65193857	0.000238205	0.003067296	*Spcs3*	0.615900138	0.006544425	0.038123042
Fmr1	0.7610213	6.02E-05	0.001031014	*Specc1l*	1.215356054	0.00020689	0.002748105
Fnip2	0.674644352	0.001974024	0.015593386	*Spg11*	0.880989816	0.001685639	0.013896043
Focad	0.741090555	0.000114335	0.001714948	*Spin1*	0.621616184	0.002830472	0.020351622
Folh1	1.16327106	3.84E-10	3.30E-08	*Spin4*	0.88692541	0.007332528	0.041381055
Foxd3	7.529418816	3.54E-05	0.000666412	*Spock1*	1.433153566	2.52E-05	0.000506275
Foxj2	0.806692672	4.74E-07	1.84E-05	*Spop*	0.433238437	0.001639396	0.013597854
Foxj3	0.746756423	3.94E-05	0.00073154	*Sppl3*	0.662817983	0.006295348	0.037017701
Foxk1	0.704577526	0.006047152	0.035968947	*Spred1*	1.145335329	0.000139934	0.002019223
Foxp4	0.780598962	0.001353004	0.011802372	*Spry1*	1.059339242	9.77E-05	0.001516573
Foxred2	1.245527059	0.00013538	0.001963994	*Spta1*	6.505306516	3.35E-05	0.000638044
Frmd5	0.69878293	1.35E-05	0.00030274	*Spty2d1*	0.771819893	6.95E-05	0.001150673
Frrs1	1.355507311	0.004980487	0.031122695	*Srcin1*	0.565573874	0.007622267	0.04261571
Frs2	0.874380797	0.000128001	0.001874202	*Srd5a1*	0.473487971	0.008692278	0.046945752
Fryl	0.60294156	0.005507858	0.03345599	*Srebf1*	0.505215798	0.008341974	0.045614211
Frzb	3.103439052	5.03E-05	0.000895121	*Srr*	0.82280894	0.001766664	0.014400709
Fubp3	0.57930863	0.000440306	0.004959766	*Ss18*	0.786481737	0.001135458	0.010378898
Fuca2	0.885231567	4.57E-05	0.000824144	*Ss18l1*	0.984665301	0.000263436	0.003317606
Furin	1.01746079	0.002049884	0.015992143	*Ssh1*	0.676812062	0.000695167	0.00706112
Fut9	1.300964889	0.005699533	0.034366274	*Sspn*	0.808622308	0.000232768	0.003009591
Fxr2	0.567573806	0.000622739	0.006478614	*Ssrp1*	0.4918462	0.005590635	0.033854486
Gaa	0.60679906	0.003704482	0.024875958	*St3gal3*	0.556592051	0.003517669	0.023875945
Gab1	0.858515081	4.42E-06	0.000121889	*St6galnac3*	0.518948494	0.003806783	0.02536469
Gab2	1.201973989	8.67E-05	0.001389936	*Stag1*	0.531193277	0.006140929	0.036355147
Gabpa	0.876996494	0.000136039	0.001970534	*Stag2*	1.134646535	5.56E-08	2.85E-06
Gak	0.750765925	0.000212635	0.002801005	*Stam2*	0.76323171	0.00038946	0.004478674
Gal3st2b	4.579710444	0.001478581	0.012578589	*Stat3*	0.486598417	0.005713529	0.034428702
Galnt1	0.57556155	0.004827078	0.030427369	*Stau1*	0.630531341	0.001880374	0.015023127
Galnt10	2.643612428	2.20E-44	2.77E-41	*Stk10*	2.309040108	7.75E-12	8.99E-10
Galnt11	0.494922826	0.000831313	0.008142124	*Stk35*	0.660378373	0.001180505	0.010692419
Galnt16	0.779752688	2.00E-05	0.000421587	*Stk4*	0.985083606	1.69E-06	5.46E-05
Galnt6	0.693400512	9.09E-05	0.001432656	*Strn*	0.84262973	2.43E-05	0.000492873
Galnt7	1.099085699	1.65E-08	9.95E-07	*Stt3b*	0.792306396	0.000530597	0.005731361
Gapvd1	0.550886019	0.009300064	0.049133345	*Stx17*	0.690525604	5.53E-05	0.000962477
Garem1	0.70388897	0.000545625	0.005856875	*Stx1b*	1.129980085	3.27E-06	9.54E-05
Gas2	0.484991204	0.006684987	0.038679617	*Stxbp1*	0.682466404	0.001883769	0.015037537
Gcc1	0.59160543	0.008326029	0.0455807	*Stxbp3*	0.45120046	0.005261785	0.032335344
Gcn1	0.777872293	3.63E-05	0.00068125	*Suclg2*	1.951945239	1.22E-12	1.57E-10
Gcnt1	1.885259267	0.005424298	0.033108009	*Suco*	0.920267333	1.02E-08	6.41E-07
Gdap2	0.486289099	0.00260024	0.019095699	*Sufu*	0.676941463	0.008228642	0.045203724
Gdpd4	4.218857779	0.000705209	0.007151587	*Sulf2*	0.731738706	7.68E-05	0.001247557
Gfra1	0.778824225	0.000747818	0.00749921	*Sun1*	0.807408506	0.001424193	0.012214843
Gid4	1.011076158	0.000272117	0.003408768	*Sun2*	1.134020539	0.000681425	0.00695142
Gk	0.708609893	0.004942483	0.030905641	*Surf6*	0.560040524	0.003038107	0.021488736
Glg1	0.608831936	0.006280089	0.036982094	*Susd6*	0.924606107	5.39E-05	0.000944762
Gli2	2.295886612	0.003215639	0.022396915	*Suv39h1*	0.502005487	0.006521759	0.03803056
Glo1	0.784818797	0.006003218	0.035809037	*Sv2a*	0.652211032	0.004865946	0.030607107
Glyr1	0.618064482	0.000765385	0.007627197	*Svip*	0.939546958	4.05E-05	0.000747472
Gna12	0.65912617	0.007571359	0.042370488	*Sybu*	0.777082905	0.000440476	0.004959766
Gnao1	0.985244499	0.001159425	0.010557136	*Sycp1*	8.228601025	4.11E-10	3.51E-08
Gnb1	0.868964459	0.000130806	0.001909367	*Syde2*	0.882575968	0.007293822	0.041205951
Gne	0.609119663	0.000760806	0.007605242	*Sympk*	0.495160075	0.000462684	0.005147804
Gng12	0.97309639	0.00014547	0.002084777	*Syngr3*	1.263290022	7.51E-05	0.00122424
Gng7	0.611753906	0.000118371	0.00176187	*Synj1*	0.875539053	0.001068997	0.0099577
Gnl3l	0.778274529	0.000259132	0.003276509	*Synj2*	0.617662922	0.000783978	0.007758917
Gnptab	1.144703091	0.000386206	0.004451493	*Synm*	0.729106257	0.000840798	0.00820951
Gns	0.991199453	6.37E-06	0.000166146	*Synrg*	0.837810064	0.000944241	0.009037461
Golga3	1.024856523	0.000187733	0.002554873	*Tacc1*	0.890628871	2.78E-07	1.18E-05
Golph3	0.622243834	0.001948299	0.015454762	*Taf1*	0.777996287	2.32E-05	0.000475602
Gosr1	0.933207416	6.57E-06	0.000169969	*Taf1b*	0.811337774	0.006738072	0.038879663
Gpalpp1	1.00901314	8.85E-07	3.14E-05	*Taf2*	0.714777902	0.003601381	0.02431318
Gpam	1.081634304	1.55E-07	7.01E-06	*Tanc1*	1.04378181	7.06E-06	0.00017917
Gpatch2l	0.555389941	0.006080862	0.036083692	*Tarbp1*	0.735045144	0.000548129	0.005872173
Gpatch8	0.721442542	0.00485623	0.030578722	*Tax1bp1*	0.695148064	6.88E-05	0.001142558
Gpd1l	0.742892275	0.002495377	0.018457389	*Tbc1d14*	0.528886224	0.002540125	0.018726979
Gpkow	0.833812999	9.93E-05	0.001534374	*Tbc1d20*	0.540918876	0.000140543	0.002021829
Gpr107	0.600956613	0.000719066	0.007265985	*Tbc1d22a*	0.557154618	0.005065278	0.031480063
Gpr146	0.59797772	0.00574834	0.034594354	*Tbc1d24*	0.862085649	0.006386348	0.037432912
Gpr75	2.144192764	2.14E-10	2.00E-08	*Tbc1d30*	1.546155197	5.19E-10	4.36E-08

Gprasp1	1.260803884	0.000222743	0.002905063	*Tbc1d5*	0.71881237	7.18E-06	0.000180996
Gprasp2	1.337650952	0.00174942	0.014290963	*Tbc1d9b*	0.713112007	0.001392733	0.012010479
Gramd4	1.081638639	0.000185412	0.002525099	*Tbccd1*	0.544340668	0.0026573	0.019394186
Grk5	0.860516313	0.006624137	0.038468835	*Tbl1x*	0.482959807	0.00236892	0.017826308
Grm3	0.734008905	0.000153812	0.002182809	*Tbl1xr1*	0.822800589	0.001950136	0.015462837
Grpel2	0.460974888	0.00438976	0.02828024	*Tbl2*	0.600512124	0.009516776	0.049984891
Gstcd	2.435312042	1.01E-28	5.76E-26	*Tcaf1*	0.843645766	0.001694652	0.013964254
Gstm1	0.762528774	1.55E-05	0.000338997	*Tcof1*	0.679425051	0.008921601	0.047774796
Gstm6	0.675038322	0.008092664	0.044720316	*Tdrd6*	1.095711326	0.007191322	0.040760886
Gtf2a1	0.865255401	4.61E-06	0.000126157	*Tdrd7*	0.66525201	0.000135116	0.001961672
Gtf2ird2	0.90418922	0.002533114	0.018689871	*Tecpr1*	0.448770505	0.003560236	0.024087022
Gtf3c1	1.028886878	0.000403188	0.004616275	*Tecpr2*	1.014116083	0.002405362	0.018028772
Gtf3c4	1.064895044	0.000126565	0.001858939	*Tedc1*	0.738947376	0.000882518	0.008528748
Gxylt1	0.904403849	0.005098253	0.031576759	*Tef*	0.582145571	0.000374386	0.004339069
Gzf1	0.613848827	0.000440995	0.004959766	*Tent2*	0.56520728	0.004532224	0.02898262
H2-Q1	7.517110871	8.97E-08	4.32E-06	*Tent5c*	1.116649491	0.008738244	0.047125088
H2-Q10	5.276753046	0.000767153	0.007636386	*Terf2ip*	0.648533158	0.003079944	0.021707751
H2-Q4	2.536448671	4.41E-06	0.000121735	*Tescl*	5.829387401	0.000171786	0.00237547
H2-Q5	4.541820938	0.001620323	0.013492932	*Tex2*	0.934237023	5.60E-07	2.13E-05
Hbs1l	0.518369003	0.001241649	0.011102599	*Tfdp2*	0.649689117	0.000566067	0.006008062
Hcfc1	0.814793168	0.000334326	0.003992269	*Tfip11*	0.527557368	0.00220318	0.016898132
Hdac11	0.708275626	0.001777888	0.014454805	*Tgfa*	0.883705173	2.26E-06	6.96E-05
Hddc3	2.216521684	5.74E-29	3.39E-26	*Tgfbr1*	0.970650179	2.86E-06	8.46E-05
Hdhd2	0.892203603	2.42E-05	0.00049094	*Tgfbrap1*	0.925813014	3.30E-06	9.59E-05
Hdlbp	0.928025292	7.28E-05	0.001193507	*Tgoln1*	0.64809368	0.007628965	0.042632926
Heatr1	0.799434954	0.003985982	0.026271621	*Thrap3*	0.951585777	6.14E-07	2.28E-05
Heatr5a	0.776236069	0.000693274	0.007049464	*Thrb*	1.483961096	0.000311157	0.003784945
Heatr5b	1.126323406	0.000280602	0.003491912	*Thsd1*	1.21396672	7.20E-07	2.62E-05
Heca	0.637462614	0.004353789	0.028117418	*Thtpa*	0.791754782	9.80E-06	0.000235598
Hecw2	0.908654723	3.75E-05	0.000700337	*Timm29*	0.696433353	0.000127144	0.001865995
Heg1	0.795899671	0.002998645	0.021269925	*Tjp1*	1.094745906	1.85E-06	5.93E-05
Hgs	0.617974581	0.002515295	0.018575777	*Tjp2*	1.010392469	6.56E-07	2.41E-05
Hhip	0.88732283	5.36E-05	0.000941569	*Tlk1*	0.774045474	0.002136291	0.016489307
Hif1a	0.866968476	4.64E-07	1.82E-05	*Tll2*	0.624327082	0.000189602	0.002567362
Hif1an	0.990525659	7.59E-07	2.75E-05	*Tln1*	1.173044324	2.85E-07	1.20E-05
Hinfp	0.612447636	0.003403064	0.023307292	*Tm9sf3*	0.815131864	7.73E-05	0.001253806
Hipk1	0.654374351	0.001869382	0.014966932	*Tm9sf4*	0.597282955	0.001945065	0.015435583
Hipk3	1.29498032	1.05E-09	8.17E-08	*Tmc7*	0.743021157	3.32E-06	9.60E-05
Hk1	0.723538027	0.00459855	0.029307616	*Tmcc2*	0.546920137	0.002048468	0.015992143
Hmga1b	3.988283118	5.28E-07	2.02E-05	*Tmed8*	1.016529658	5.87E-07	2.20E-05
Hmgxb3	1.006842019	0.000282456	0.003501158	*Tmem106b*	1.173853636	1.31E-08	8.10E-07
Hmgxb4	0.698227018	0.000362093	0.004227698	*Tmem123*	0.558287784	0.000531864	0.005741761
Hnrnph2	0.607140596	0.008960503	0.04795293	*Tmem127*	0.774901134	0.002354639	0.017747104
Hnrnpll	0.564283003	0.00851977	0.04630512	*Tmem131*	1.003971453	0.006982778	0.03987778
Hnrnpr	0.482722487	0.003487666	0.023755907	*Tmem132b*	0.995776555	4.32E-06	0.000120035
Hnrnpu	0.609759246	0.001344551	0.011765922	*Tmem132c*	2.738708069	1.07E-05	0.000251136
Hps5	0.681208823	0.001176614	0.010672524	*Tmem144*	0.629689361	0.004036427	0.026520884
Hr	0.91795243	3.42E-05	0.000647543	*Tmem150c*	3.27866125	2.65E-10	2.35E-08
Hs2st1	0.614098752	0.004121148	0.02696057	*Tmem151a*	0.534548512	0.000836334	0.008182827
Hs6st1	1.006990862	1.43E-06	4.74E-05	*Tmem161a*	0.610563803	0.003110114	0.021855197
Hsp90b1	0.683479903	0.000667368	0.006837538	*Tmem167b*	0.64458639	0.00179736	0.014569249
Hspa12a	0.700012838	0.003400743	0.023299838	*Tmem168*	0.630058598	0.006890731	0.039531254
Hspa4	0.810325561	4.11E-07	1.64E-05	*Tmem170b*	1.013033798	0.001247671	0.011123488
Hspa4l	1.100340428	6.59E-06	0.000170092	*Tmem181a*	0.563310141	0.005742368	0.034569419
Hspa8	0.63026425	0.003870439	0.025689221	*Tmem184b*	0.899495383	2.60E-06	7.79E-05
Hspa9	0.807139571	4.33E-05	0.000784609	*Tmem201*	0.632482587	0.000220214	0.002880764
Hspg2	1.66080045	7.95E-12	9.17E-10	*Tmem229a*	0.931721029	0.000307065	0.003752068
Hsph1	0.705702554	0.006285278	0.037001127	*Tmem245*	0.636479667	0.005486886	0.0333593
Htra1	1.162565569	0.001368876	0.01185944	*Tmem263*	0.675422794	0.000243154	0.003114052
Hunk	0.910827508	3.77E-07	1.52E-05	*Tmem30a*	0.784042677	0.000111526	0.001681972
Hus1	0.854616374	1.40E-05	0.000312694	*Tmem56*	0.870891388	0.000103746	0.001593103
Hyou1	0.67358837	0.002990313	0.021234368	*Tmem8b*	0.742380337	0.002296378	0.017377275
Iars2	0.510218881	0.007023537	0.040050053	*Tmem94*	0.897441966	0.003172149	0.022203686
Ibtk	0.900721293	2.30E-05	0.000472639	*Tmod2*	0.632130572	0.002770986	0.020023848
Icmt	1.110577287	1.62E-07	7.29E-06	*Tmpo*	0.600988195	0.004191347	0.027339701
Ids	0.629048911	0.002809412	0.020215574	*Tmtc2*	0.637172173	0.001607505	0.013412599
Ifit2	1.144835321	1.15E-06	3.96E-05	*Tmtc3*	1.195685743	6.28E-05	0.001067668
Ifnar1	0.704650652	0.000281944	0.003499316	*Tmx3*	0.618466409	0.003918131	0.025878558
Ift122	0.705751319	0.003837617	0.025507198	*Tmx4*	0.937114721	1.25E-06	4.23E-05
Ift140	0.764998581	0.000285613	0.003533335	*Tnfrsf19*	1.190409752	0.000386926	0.004457076

Ift80	0.877226091	0.000149918	0.002135573	*Tnfsf9*	0.754135078	0.001317804	0.011590277
Igf2r	1.406735556	1.16E-05	0.000267929	*Tnks*	1.38266153	2.92E-07	1.22E-05
Igsf11	0.710842619	0.001037448	0.0097228	*Tnks1bp1*	1.309790374	6.17E-08	3.10E-06
Ikbkg	0.761784674	0.000178831	0.00245315	*Tnks2*	0.796068303	0.002939049	0.020956942
Il12rb1	1.184232065	0.002160366	0.016654728	*Tnn*	7.176997673	8.17E-13	1.07E-10
Il13ra2	2.188866577	0.004618113	0.029405713	*Tnpo1*	0.974748307	2.83E-05	0.000554563
Il17rb	0.92680207	2.43E-06	7.41E-05	*Tnpo2*	0.561267107	0.001808391	0.014633568
Il1rap	0.889898582	3.05E-05	0.000590902	*Tns1*	1.607693455	1.72E-10	1.62E-08
Il3ra	3.205091162	2.89E-05	0.000565521	*Tns3*	0.907804038	0.001710902	0.014049574
Il6st	1.006691938	2.03E-05	0.000427697	*Tollip*	0.721188049	0.000140349	0.002020732
Ilf3	0.589966154	0.000270033	0.0033849	*Tom1l2*	0.984396895	2.77E-05	0.000545228
Immt	0.378708781	0.008551723	0.046388347	*Tomm70a*	0.588115095	0.001280248	0.01135109
Impact	0.497257727	0.000891569	0.008603027	*Top2b*	1.019140502	8.76E-05	0.001401121
Impg2	3.442905162	0.002495413	0.018457389	*Topbp1*	0.832473176	0.000553767	0.005916811
Inip	0.481651673	0.003427029	0.023412047	*Tor1aip1*	0.708766027	2.38E-05	0.00048459
Ints1	0.803110646	0.00031295	0.003801855	*Tox3*	2.574174943	5.06E-18	1.21E-15
Ip6k1	0.749965446	0.006930128	0.039683879	*Tpd52*	0.366220868	0.007816696	0.043496909
Ipmk	0.899638037	6.99E-07	2.56E-05	*Tppp*	1.285705441	7.68E-06	0.000190584
Ipo13	0.461863301	0.001933697	0.015358268	*Traf6*	0.993190212	4.33E-06	0.000120077
Ipo7	0.667109501	0.003237692	0.022509041	*Trappc12*	0.586880017	0.005526346	0.033503695
Ipo9	0.961121197	8.15E-08	3.96E-06	*Trappc8*	0.943731812	7.80E-05	0.001262747
Iqcf3	2.840437465	1.18E-06	4.03E-05	*Trim17*	0.942311457	0.003759205	0.025136276
Iqub	2.928871862	0.00339437	0.0232646	*Trim25*	0.747909817	0.001108613	0.010212528
Ireb2	0.775362663	0.001243043	0.011105096	*Trim28*	0.466717353	0.000949958	0.009087581
Irgq	0.7822097	0.008185791	0.04506599	*Trim36*	0.561631118	0.001063664	0.009924204
Irs1	1.00864874	0.004230425	0.027508679	*Trim44*	1.33604462	3.39E-06	9.74E-05
Isoc1	0.571783206	0.004578163	0.029197371	*Trip12*	0.683795444	0.000362016	0.004227698
Itch	0.980800717	1.82E-06	5.85E-05	*Tro*	1.151911664	0.001360038	0.011822659
Itgav	1.183741721	1.07E-05	0.000251136	*Trp53bp1*	0.84448203	0.008965014	0.04795293
Itgb3	0.800102615	5.60E-05	0.000973172	*Trp53inp1*	0.70639775	0.000132439	0.001927243
Itgb4	0.570495394	0.000761701	0.007606143	*Trpc1*	0.640834737	0.003214848	0.022396915
Itgb8	1.099831377	2.03E-06	6.41E-05	*Tsc2*	0.677649099	0.0007877	0.007791675
Itih5	1.901818138	0.002217349	0.016948866	*Tspan2*	0.577775437	0.008209801	0.045139577
Itpk1	0.630242437	0.005982846	0.035701792	*Tspyl4*	0.807801308	7.10E-06	0.000179995
Ivns1abp	0.521989078	0.004571342	0.029163711	*Tsr1*	0.850062957	0.001818877	0.014686986
Jarid2	0.78429608	0.000821868	0.008070533	*Ttc17*	1.039946667	0.001345086	0.011765922
Jmjd4	0.803282549	0.008803663	0.0473175	*Ttc37*	0.961283372	1.06E-05	0.000249875
Josd1	0.562529013	0.001454326	0.012439423	*Ttc7*	0.618288221	0.003044452	0.021497674
Jph1	0.674905879	0.000521457	0.005661752	*Ttc9c*	0.529711703	0.008185394	0.04506599
Jup	0.635640909	0.003561943	0.024089952	*Ttll7*	0.705513305	0.004595544	0.029298334
Kansl1	0.71570517	0.003284091	0.02271545	*Tubgcp5*	0.58622094	0.004730972	0.029979743
Kansl1l	0.469072611	0.004288676	0.027810922	*Tubgcp6*	0.774740838	0.008644596	0.0467273
Kantr	1.196968772	2.73E-05	0.000539469	*Tug1*	0.626517402	0.000309076	0.003766902
Kbtbd8	0.860947273	0.001865966	0.014955827	*Tulp4*	0.525439258	0.001634193	0.013560642
Kcna6	1.005392478	2.30E-05	0.000472905	*Tut7*	0.578226453	0.002989229	0.021234368
Kcnh7	1.074243281	1.63E-06	5.29E-05	*Twsg1*	0.662237575	0.00230079	0.017396733
Kcnj10	3.06344049	8.01E-48	1.08E-44	*Txndc16*	0.610690969	0.00052791	0.00571867
Kcnj2	0.80808241	0.008350428	0.045620851	*Txnrd1*	0.792609017	0.00047121	0.005224208
Kcnma1	2.590728299	0.000612355	0.006381116	*Tyro3*	0.599391348	0.000298037	0.003667839
Kctd20	0.529402873	0.001708789	0.014049574	*Ubap2*	0.661983374	0.00503856	0.031371511
Kdm2a	0.429687163	0.003316785	0.022882182	*Ube2h*	0.578176607	0.001448565	0.012395759
Kdm3a	0.693312529	0.006328984	0.037142746	*Ube2o*	0.603077634	0.000490784	0.005409493
Kdm4b	0.714646385	0.000989934	0.009359735	*Ube3b*	0.855336232	1.19E-05	0.000272491
Kdm4c	0.703538267	0.000440851	0.004959766	*Ube4a*	0.881714153	4.78E-06	0.000130257
Kdm7a	0.769598845	0.000252183	0.003212279	*Ube4b*	0.445554466	0.009520742	0.049991832
Keap1	0.446664882	0.007820885	0.043507414	*Ubiad1*	0.696190609	0.005831718	0.035018095
Khnyn	0.786798461	1.19E-05	0.000271706	*Ubqln1*	1.021365546	5.86E-06	0.000155043
Khsrp	0.871599274	8.96E-05	0.001423031	*Ubr2*	0.509910661	0.004997041	0.031183647
Kidins220	0.956640628	0.000564607	0.00599818	*Ubr3*	0.744729261	0.000493803	0.005430114
Kif13a	0.964831449	9.12E-05	0.001436071	*Uck2*	0.614384969	0.00073527	0.007408749
Kif13b	1.187257896	6.97E-06	0.000177033	*Ufl1*	0.491246457	0.002261574	0.017182286
Kif16b	0.837315243	0.000548881	0.005875143	*Ugcg*	0.820009764	0.000749379	0.007510876
Kif1a	0.867904856	8.22E-06	0.000201451	*Ugt8a*	0.779235511	0.000414061	0.004720742
Kif1b	1.199085285	1.35E-06	4.50E-05	*Uhmk1*	1.453758538	2.75E-08	1.55E-06
Kif3c	0.889282726	2.30E-05	0.00047327	*Uhrf1bp1*	1.386328404	2.76E-08	1.55E-06
Kl	1.496371842	0.000634174	0.006579472	*Uhrf1bp1l*	0.501780205	0.003087	0.021741268
Klc3	3.103024139	0.000636028	0.00659147	*Ulk1*	0.607329334	0.004733351	0.029984766
Klhdc10	0.833381436	4.07E-05	0.000749203	*Ulk2*	0.983371311	0.000189507	0.002567362
Klhl11	0.959973952	0.001164249	0.01059086	*Unc119b*	0.515560842	0.009456699	0.049780002
Klhl2	0.703742448	0.001297196	0.011451557	*Urb2*	0.962299444	0.000823767	0.008080779

Klhl22	0.57903702	0.001872851	0.014982015	*Uri1*	0.645663686	0.000309849	0.003773885
Klhl24	0.941100021	2.93E-05	0.000570976	*Uso1*	0.608058035	0.007187901	0.040756781
Klhl33	4.118835345	1.99E-08	1.17E-06	*Usp10*	0.801796893	7.16E-06	0.000180687
Klhl4	0.649664511	0.001041368	0.009754701	*Usp11*	0.544867529	0.005910654	0.035402122
Klhl42	0.869105899	0.00709542	0.040400828	*Usp22*	0.70101686	1.71E-05	0.000368643
Klhl7	0.474727398	0.007492437	0.042026568	*Usp25*	0.519166729	0.006705247	0.038784973
Klrg2	1.338366524	1.78E-05	0.000380861	*Usp30*	0.407321758	0.00714732	0.040633318
Kpna1	0.807578955	0.001983584	0.015626048	*Usp31*	0.900556412	0.001247478	0.011123488
Kpna4	0.442777058	0.003886508	0.025750672	*Usp32*	0.859396458	0.000571434	0.006046607
Kpna6	0.65469381	0.00108291	0.010034435	*Usp33*	1.040573429	4.73E-05	0.000848783
Krr1	0.601703081	0.000202659	0.002707323	*Usp38*	0.646736142	0.003246678	0.022530083
Ktn1	0.896716291	3.25E-05	0.000621051	*Usp42*	0.628331463	0.000320623	0.003867727
L3mbtl2	0.513870317	0.008348413	0.045620851	*Usp45*	0.920460901	0.009446797	0.049769453
Lamc1	1.018692793	0.000105417	0.001610918	*Usp46*	0.615559512	0.002030961	0.015897001
Larp4b	0.62421853	0.001983945	0.015626048	*Usp47*	1.006001315	5.27E-06	0.000141793
Lars	0.552723062	0.000302089	0.003705636	*Usp54*	1.259022932	0.000659693	0.006777265
Lats1	0.975327603	3.53E-06	0.000100698	*Usp6nl*	0.925346391	2.88E-06	8.50E-05
Lats2	1.057465727	5.00E-05	0.000891741	*Usp8*	0.877423726	0.00172205	0.01411618
Lcorl	0.617704749	0.001867496	0.014958171	*Usp9x*	1.316166515	6.04E-08	3.06E-06
Ldlr	0.995593503	8.67E-05	0.001389936	*Utp15*	0.558970347	0.001626293	0.0135167
Ldlrad3	0.686402012	0.000825989	0.008094179	*Vcpip1*	0.624356009	0.003916951	0.025878558
Lemd3	0.865326227	1.67E-05	0.000361932	*Vegfb*	0.444869321	0.007095739	0.040400828
Lepr	3.250941388	0.009171298	0.048739119	*Vezf1*	0.923330753	1.03E-06	3.60E-05
Leprotl1	1.300130278	1.13E-18	3.01E-16	*Vkorc1l1*	0.785810849	7.67E-05	0.001246948
Lgals8	0.441064003	0.004371975	0.028205954	*Vps11*	0.592800688	0.000300014	0.003687362
Lgalsl	0.758039037	0.002733351	0.019826756	*Vps33a*	0.806642356	2.27E-06	6.99E-05
Lgr4	0.898636789	0.003537157	0.023973786	*Vps37a*	0.651839325	0.001597198	0.013363702
Lhfpl4	0.87800476	0.000443615	0.004979609	*Vps39*	0.707095821	0.002799396	0.020174224
Lig3	0.776170549	0.000330733	0.003954364	*Vps4b*	0.509050889	0.003040414	0.021490559
Lin52	0.593890497	0.00181807	0.014686739	*Vps8*	0.838855136	0.004617192	0.029405713
Lin54	0.692169883	3.12E-05	0.000599806	*Vwa1*	1.066275797	0.000426451	0.004844477
Lin7c	0.829852157	3.34E-06	9.64E-05	*Wac*	0.765881424	5.14E-05	0.000908932
Lingo3	1.266520204	0.001243103	0.011105096	*Wars*	0.601683811	0.002923426	0.02087704
Llgl1	0.473775624	0.008622855	0.046650781	*Wasf1*	0.478554971	0.002126011	0.016416661
Lmbrd1	0.71338414	0.000351499	0.004147773	*Wasf3*	0.875686474	0.001892812	0.015084246
Lmtk2	1.166359146	3.07E-06	9.01E-05	*Washc2*	0.607890686	0.002468202	0.018354216
Lonrf1	0.654580303	0.009053167	0.048274193	*Washc5*	0.67044008	0.002457703	0.018299539
Lpgat1	0.548722477	0.0067259	0.038833137	*Wbp1l*	0.784011782	0.001240228	0.011095141
Lpin1	0.667819625	0.00034479	0.004083688	*Wdfy1*	2.504503797	2.40E-35	2.16E-32
Lpin2	0.890027047	0.000253241	0.003220807	*Wdfy2*	1.13434275	0.000646414	0.006677136
Lratd2	1.979103448	3.34E-10	2.91E-08	*Wdr11*	0.790548511	0.001401082	0.012065897
Lrch1	0.986595853	1.02E-05	0.00024338	*Wdr19*	0.872828417	0.008410657	0.045876799
Lrp6	1.024756278	9.44E-05	0.001473248	*Wdr25*	0.623765327	0.005112113	0.031652235
Lrpprc	0.589721819	0.003881135	0.025733109	*Wdr37*	0.608513334	0.000366727	0.004265999
Lrrc1	0.642070631	0.00220891	0.01690487	*Wdr43*	0.443441707	0.00513865	0.031788944
Lrrc47	0.531149862	0.00185902	0.014917039	*Wdr46*	0.508761494	0.008283929	0.045401888
Lrrc58	0.701211473	0.002887479	0.02066718	*Wdr47*	0.604493843	0.006798713	0.039141318
Lrrc59	0.497510788	0.003987536	0.026272707	*Wdr48*	0.804004067	9.97E-05	0.001536168
Lrrc66	5.533913209	4.64E-08	2.46E-06	*Wdr7*	1.072312556	7.41E-06	0.000184843
Lrrc8d	0.878395021	0.000768644	0.007647201	*Wdr81*	0.76856601	4.92E-05	0.000877798
Lrrn1	0.841240621	4.32E-05	0.000783627	*Wipf1*	0.842791084	0.003041171	0.021490559
Lrrn3	1.193849338	0.0004655	0.005174879	*Wipi2*	0.538282061	0.000384636	0.004436107
Lrtm2	0.829794334	0.001386104	0.011964169	*Wiz*	0.525621891	0.003299234	0.022788388
Lsm14a	0.686872678	2.47E-05	0.000499516	*Wnk3*	1.31475849	0.001129311	0.010347733
Lsm14b	0.576118523	0.001197357	0.010782652	*Wwp2*	0.525015358	0.007220856	0.040879257
Ltbp2	3.990384793	0.0048844	0.030642912	*Xkr8*	1.077750664	2.10E-07	9.11E-06
Ltn1	0.522134716	0.005300203	0.032518575	*Xpo1*	0.860201119	0.000381478	0.004410447
Lurap1	0.778644324	0.004384953	0.028270383	*Xpo7*	1.069442598	3.39E-06	9.74E-05
Luzp1	0.831311802	0.006541869	0.038119897	*Xpot*	0.954004526	1.45E-05	0.000321373
Luzp2	1.792506622	3.98E-12	4.85E-10	*Yes1*	0.834424581	0.000277293	0.003461903
Lysmd3	0.674057812	0.001830098	0.014752384	*Yipf6*	1.086756882	7.59E-06	0.000188476
Maco1	0.606149565	0.003177613	0.022230357	*Ypel2*	0.435377506	0.001231771	0.011029923
Magt1	0.771589739	0.00077332	0.007689674	*Ythdf3*	0.961389388	1.47E-05	0.000325351
Man2a2	1.322806912	2.78E-07	1.18E-05	*Ywhag*	0.608394044	0.002864927	0.020529079
Man2b1	0.936193475	5.53E-09	3.78E-07	*Zbtb1*	0.532696926	0.00504477	0.031399832
Manba	0.671115863	0.001075957	0.009987248	*Zbtb11*	0.526965635	0.00794135	0.044022092
Manea	0.740496913	0.001478569	0.012578589	*Zbtb14*	0.590927799	0.002046916	0.015988784
Map2k4	0.481197303	0.000657662	0.006763758	*Zbtb18*	0.58008297	0.003679	0.024748802
Map3k11	0.665597804	1.89E-05	0.000403278	*Zbtb34*	1.038052307	0.00023418	0.003025766
Map3k13	1.133031947	2.39E-06	7.31E-05	*Zbtb38*	0.529405565	0.000868578	0.008424184

Map3k4	0.524022369	0.001623258	0.013505475	*Zbtb41*	1.349482376	9.09E-10	7.13E-08
Map3k7	0.859537666	0.002409502	0.018052645	*Zbtb44*	1.050390608	2.51E-05	0.000505917
Map4k5	0.825936615	5.23E-05	0.00092192	*Zbtb6*	0.820521618	0.000674414	0.006892398
Map6d1	0.706168698	5.76E-05	0.000994211	*Zcchc24*	1.066563028	9.95E-06	0.000238151
Mapk14	0.455377626	0.002948412	0.02099994	*Zdhhc17*	0.658544581	0.001217051	0.010913618
Mapk8ip3	0.924271033	4.19E-05	0.000766337	*Zdhhc18*	0.953559317	0.001966335	0.015561168
Mapk9	0.617022397	0.008059717	0.044573676	*Zdhhc2*	1.686208323	6.97E-15	1.19E-12
Marchf8	0.835803903	4.52E-06	0.000124212	*Zdhhc21*	0.673657273	0.000473618	0.005242273
Marveld2	2.091179646	0.005628453	0.034024512	*Zdhhc3*	0.835015915	1.72E-05	0.000369426
Mast4	0.59697851	0.005091918	0.031558203	*Zdhhc5*	0.749413036	8.21E-05	0.001322259
Matn2	0.725274331	5.96E-05	0.0010241	*Zdhhc8*	0.427311612	0.003578131	0.024190775
Matr3	0.613563034	0.000156477	0.002210675	*Zeb2*	0.797191646	0.002392868	0.017963613
Max	0.507811289	0.000572493	0.006052483	*Zer1*	0.615520764	0.000669772	0.006858455
Mb21d2	0.531913069	0.006653876	0.038594115	*Zfp105*	0.58799026	0.00849935	0.046242688
Mbd5	0.601227449	0.008767314	0.047229564	*Zfp106*	1.357994383	3.83E-05	0.000712099
Mbnl1	0.686797243	0.000710171	0.007191402	*Zfp110*	0.478364045	0.002235555	0.017032931
Mboat4	5.648176288	0.006718373	0.038815083	*Zfp12*	0.608237802	0.009108772	0.048474976
Mbtps1	0.813472831	7.43E-05	0.001213504	*Zfp143*	0.705580888	5.21E-05	0.000918938
Mcm3	0.587867979	0.005114782	0.031658393	*Zfp146*	0.893548898	1.41E-06	4.69E-05
Mcm4	0.898068044	0.000595939	0.006251405	*Zfp148*	0.585552391	0.001703672	0.014017681
Mcm7	0.620575111	0.001561713	0.013161418	*Zfp174*	1.14817077	0.007455005	0.041878739
Mcm9	0.696788263	0.002581062	0.018969603	*Zfp180*	0.798789173	0.002672434	0.01948959
Mdc1	1.68917181	3.81E-09	2.70E-07	*Zfp189*	0.539163785	0.007838683	0.04356796
Me1	0.625260224	0.009361239	0.049373744	*Zfp2*	0.697995909	1.22E-05	0.000277637
Mecp2	0.478041933	0.006904586	0.039586711	*Zfp251*	0.679901355	3.38E-05	0.00064124
Med14	0.639493479	0.000763627	0.007621351	*Zfp26*	0.646035643	0.003284765	0.02271545
Med20	0.533495226	0.00157181	0.01322882	*Zfp260*	0.484115695	0.003698507	0.024844661
Med24	0.691316717	0.005921125	0.035453602	*Zfp275*	1.014304252	6.28E-05	0.001067668
Mef2a	0.650554261	0.001286516	0.011392293	*Zfp282*	0.424735359	0.007835765	0.04356796
Mef2b	2.106651093	7.73E-07	2.80E-05	*Zfp292*	0.881178453	0.002314597	0.017480154
Megf10	1.245268668	6.73E-07	2.47E-05	*Zfp316*	0.769459177	0.000632016	0.006560675
Mex3c	0.754376077	0.001514843	0.012846606	*Zfp317*	0.471625769	0.003590735	0.024258639
Mfap1a	0.596336015	0.001986681	0.015634564	*Zfp319*	0.860337673	9.83E-06	0.000235952
Mfap3l	1.382859886	1.64E-06	5.32E-05	*Zfp334*	1.052422169	3.80E-07	1.53E-05
Mfhas1	1.530074965	2.59E-06	7.76E-05	*Zfp354c*	0.775596402	0.004987592	0.031146496
Mfn1	0.391255165	0.007447909	0.041857353	*Zfp365*	0.789633692	0.001744118	0.014259975
Mfn2	0.552295316	0.001182062	0.010701394	*Zfp37*	0.666738706	6.55E-05	0.001095028
Mfsd1	0.432939083	0.006716445	0.038815083	*Zfp39*	1.373994034	1.57E-08	9.53E-07
Mfsd2a	0.674589004	0.003891854	0.025777059	*Zfp397*	0.627864282	0.004774967	0.030177603
Mfsd4a	1.315007109	1.58E-05	0.000343514	*Zfp398*	0.567703444	0.009031349	0.048225875
Mfsd6	1.170038641	0.000500768	0.005481193	*Zfp41*	0.586087187	0.008594441	0.046563689
Mgat3	0.833323665	2.03E-05	0.000427697	*Zfp422*	0.606420908	0.00294641	0.020993588
Mgat4a	0.942424178	0.003253986	0.022564232	*Zfp438*	0.621579596	0.002853496	0.020485999
Mgrn1	0.382211585	0.009515236	0.049984891	*Zfp449*	0.586761389	0.005632317	0.034036982
Mib1	0.969004458	0.001096952	0.010129793	*Zfp46*	0.503430343	0.009494879	0.049923324
Mid1ip1	0.743296657	0.00016268	0.002271159	*Zfp507*	0.723159891	0.000273881	0.003428591
Mid2	0.850410062	0.009322417	0.049223922	*Zfp51*	0.77574753	0.003152409	0.022103109
Miga1	0.75933645	7.14E-06	0.000180341	*Zfp516*	1.140962813	0.001877043	0.015002853
Mkrn1	0.481232616	0.008505755	0.046242688	*Zfp518a*	0.843957447	9.81E-05	0.001520618
Mlec	0.962925917	1.47E-05	0.00032511	*Zfp526*	1.528419049	8.85E-10	6.97E-08
Mllt1	0.61402085	0.006290571	0.037009232	*Zfp553*	0.611931017	0.001050209	0.00981805
Mmp17	2.105605854	0.001248693	0.011123488	*Zfp568*	1.040403778	2.02E-06	6.38E-05
Mnt	0.868402509	0.000567705	0.006015323	*Zfp592*	0.866753743	0.000161435	0.002257111
Mob1a	0.757388002	1.44E-05	0.00032014	*Zfp605*	0.701719287	0.002989896	0.021234368
Mob1b	0.985404631	2.45E-05	0.000496033	*Zfp619*	1.004012127	0.001026775	0.009646685
Mon1b	1.124150876	2.94E-07	1.22E-05	*Zfp629*	0.976079223	0.00026983	0.0033846
Mon2	0.785721626	0.000730909	0.007370445	*Zfp638*	0.908525041	0.000292216	0.003604625
Morc2a	0.720908134	0.006735574	0.038877116	*Zfp646*	0.963119433	0.000312323	0.003796688
Morc4	1.110349463	8.98E-05	0.001423031	*Zfp651*	0.779555478	0.000308403	0.003763553
Mpp2	0.698913754	0.001210086	0.010866629	*Zfp654*	0.946312429	4.29E-05	0.00077869
Mpp5	0.58665258	0.002780451	0.02007629	*Zfp658*	1.266045967	4.15E-08	2.22E-06
Mras	0.689407375	0.003293759	0.022764875	*Zfp664*	0.695538594	0.000516702	0.005626274
Mrs2	1.139726298	0.00010497	0.001605372	*Zfp667*	0.78024074	0.003201036	0.022319876
Msh6	0.782921017	0.000289521	0.003577001	*Zfp687*	0.517547308	0.000535058	0.005769657
Msl2	0.881385831	4.19E-08	2.23E-06	*Zfp709*	0.819952941	0.002084098	0.016195163
Mtcl1	1.12340402	0.001421925	0.012202314	*Zfp738*	0.590588095	0.005228419	0.032203588
Mthfd1	0.723569959	0.000305069	0.003734923	*Zfp74*	0.990248834	0.000188055	0.002555573
Mtmr1	0.693354655	0.000519361	0.005648723	*Zfp758*	0.747035253	0.009220556	0.048890929
Mtmr10	1.203395709	8.70E-09	5.57E-07	*Zfp84*	1.086191456	1.49E-05	0.000329049
Mtmr3	0.995533485	2.99E-08	1.66E-06	*Zfp874a*	0.602092461	0.000203148	0.002711935

Mtmr4	0.812348998	0.001820032	0.014690033	*Zfp91*	0.625054636	0.000211932	0.002793689
Mtmr6	0.636966781	0.009186615	0.048806797	*Zfp933*	1.352761898	8.47E-10	6.73E-08
Mtmr9	0.62231432	0.006203515	0.036633876	*Zfp943*	0.527813954	0.002208215	0.01690487
Mtor	0.748592671	0.000655819	0.0067544	*Zfp953*	0.867619616	0.001906639	0.015181636
Mtrex	0.75981622	0.00139274	0.012010479	*Zfp955b*	0.560194647	0.002471652	0.018365425
Mturn	1.228648765	7.83E-06	0.000193456	*Zfp960*	1.574405535	0.000877611	0.008499731
Mtus1	0.832543119	0.002369998	0.017827326	*Zfp979*	1.622243211	0.001107061	0.010203202
Mxd1	0.486586727	0.000537955	0.005790984	*Zfp992*	3.974343692	2.81E-12	3.45E-10
Mxi1	0.516856898	0.003369209	0.023150913	*Zfx*	0.744334256	0.000641617	0.006634899
Myh14	0.778467727	0.00309463	0.021786885	*Zfyve1*	0.478671111	0.006830571	0.039269548
Myh3	2.269862668	0.000140678	0.002022237	*Zfyve26*	0.766179081	2.47E-05	0.000499516
Myh9	0.892953583	3.84E-05	0.000714159	*Zfyve27*	0.469254717	0.001432483	0.012280373
Myo18a	0.837037062	0.000453644	0.005075953	*Zhx1*	1.010792441	0.001353351	0.011802372
Myo1e	0.912610495	0.002909548	0.02079569	*Zik1*	0.954791517	0.002546946	0.018762638
Myo6	0.645779715	0.000154296	0.002186394	*Zkscan1*	1.110999075	2.74E-08	1.55E-06
Myorg	0.691042804	0.008093329	0.044720316	*Zkscan17*	0.626678843	0.00226243	0.017182286
Myrf	0.865370512	7.22E-06	0.000181667	*Zkscan5*	0.579790523	0.001668962	0.013788628
N4bp2	0.676485261	0.007532815	0.042215475	*Zkscan8*	1.037881533	1.33E-07	6.08E-06
Naa15	0.615593222	0.000525493	0.005696481	*Zmiz1*	0.788524659	0.007860986	0.043666243
Naa30	0.70811594	0.000194001	0.002617555	*Zmym2*	0.702016667	0.001534122	0.012980981
Nab1	0.924806095	0.000330547	0.003954364	*Zmym4*	0.708902681	0.000674546	0.006892398
Nacad	0.749871723	0.004398556	0.028309809	*Znfx1*	0.907479835	1.84E-05	0.000392443
Nacc1	0.855424148	6.52E-06	0.00016901	*Zpld1*	7.352414438	5.52E-06	0.000146844
Nacc2	0.621977738	0.003976657	0.026219307	*Zranb3*	0.736608283	0.004093057	0.026799812
Nadk	0.579630972	0.002668385	0.019467576	*Zrsr1*	3.008359648	4.42E-49	7.60E-46
Nalcn	0.734565348	0.004278798	0.027766067	*Zswim4*	0.553749786	0.009223506	0.048892858
Nbas	0.986273116	2.36E-10	2.14E-08	*Zswim5*	0.792789322	0.001975936	0.015595458
Nbr1	1.017684202	3.56E-07	1.45E-05	*Zyg11b*	1.017152454	4.65E-07	1.82E-05
Ncam1	0.655126993	0.000110482	0.001673425	*Zzz3*	0.965643376	0.003248626	0.022535331

Supplementary Table 1. List of 1,726 gene transcripts upregulated in adult OLs compared to adult OPCs (log2Foldchange in aOLs compared to aOPCs reported the second column, cut-off pvalue < 0.01 and qvalue < 0.05) and with at least one hyper-hydroxymethylated CpG at the promoter or gene region. Solute carrier family members genes are highlighted (bold typo, grey background cell).

Supplementary Table 2

Gene	Forward primer	Reverse primer
Abtb2	ACCGAGTCCATGGAAATCCC	CAGGGCATCTAGCTGGAACA
Adam17	CTGCTTCCAAAAGGGCTCATA	AGGTTCAGCTCGTCTCTTCA
App	GAACTTGCCCAAAGCTGACAA	ATTGGCTGCTTCCTGTTCCA
Celsr2	AGCAATGACTGGGACAGCTA	TGGGTTCAGGTCACACACA
Chd4	GCAGACAGCAGTCTTCCTCTA	GACAGAGGAGCGCTCACTAA
Clic5	ACGGACTCAGCGACAACTAA	ACAGTTGCCAATGCTTTCCC
Cpd	TGATAACCCAGGTGTCCATGAA	AGCTCTCTTCCAACCACTTCA
CSPG4	GCAGAGGAGGTCTTGGTGAA	GAGGACATCTCGTGCTCATACA
Dlg2	GGTCCCTTCGAACCAATCAGAA	CCACTGTCCTTGCTCTTGTCA
Dnmt1	AGCCATTGGCCTGGAGATTA	GCAGCCTCCTCTTTTGCTTTA
Dnmt3a	CGCCAGAAGTGCAGAAACA	AATGAAGAGTGGGTGCTCCA
Elf1	CCAGAGCAGCGCAAGAGAAA	TTGGTGTAGTCGTCGGGGAA
Enpp6	AGCCAGGAGGAAAGTCTACA	GGCAGTAAGTTGGTCTGACA
Ezh1	TCTGGGGACAAAGACATGCA	GCTCATCTGTTGGCAGCTTTA
Gapdh	CAAGGTCATCCCAGAGCTGAA	CAGATCCACGACGGACACA
Gnptab	AGAACACAACCGAACCGACAA	AGCATGGGCACCTTAATGCA
Gpr17	CCCAGTCTCCTGTCCTTTCC	GTAGGGCTGCCTCCAGAC
Gpr37	CCTGCAAGATCGTGCCCTATA	ACAGTGCACATAAGGTGAAGGTA
Hdac1	CCGAAGAGAGTGGGGATGAAG	TCACAGGCAATGCGTTTGTC
Heg1	GCCCGGTTATATCCGAACTACA	CAGGGCAAAGGTGGTTTTCA
Hes5	AAGAGCCTGCACCAGGACTA	GTGCAGGGTCAGGAACTGTAC
Hipk3	CGGTTGTGGAGATGCTTTTCA	GTTTACCATGTGCTGCAGGAA
Id2	ACCCTGAACACGGACATCA	TCGACATAAGCTCAGAAGGGAA
Id4	CACTCACCGCGCTCAAC	CGGCAGAGAATGCTGTCAC
Igf2r	TCAACGATTCTGCTCAAGGAC	CAGGCATTGCACCACAGATA
Il17rb	TGGCAGCTGGGATCTACCTAA	GGCAGGAGCATGGTGGAAATA
Kcna6	CTAGCATCCCAGAGGAGCAA	TCGGGTACTCTTTGGATGCA
Lpin1	CTCTCTTGTCCAACGTCAGCTA	ATCATTGGGGCCGTTCTTCA
Mag	CAGAGAGCCACTGCCTTCAA	CTGCACAGTGTGACTCCAGAA
Mbp	CCCTCACAGCGATCCAAGTA	CTCTGTGCCTTGGGAGGAA
Mfsd6	TTCTCCTCTTTGCCCTGATCC	TGGGATCCTTTCTGCCAACA
Mobp	CAGGCTCTCCAAGAACCAGAA	TCACGCTTGGAGTTGAGGAA
Mog	ACACCTGCTTCTTCAGAGACC	CCGGGGTTGACCCAATAGAA
Mtmr10	AAGTACCCGGTGGTTAGAGTAC	GAGACGCTGGCTTTCAAGAA
Myrf	GCTGGAGAATTCCATGCCTA	ACAGGGATGTGGTAGGTGAA
Ncoa2	GTGTCATCGACAAGGATGCA	CCTTCCAGGTTCACAACGAA
Ndrg1	ACATGAACCCGAGCAACCTA	CATTGGCCGCTCAATCTCC
Ndrg2	CAAGCTGACAGAGGCTTTCAA	ATAGGCGAGTCATGCAGGAA
NKX2-2	CCAGCCTCATCCGTCTCA	CGGGCACGTTTCATCTTGTA
Olig1	CTGTATGAGCTGGTGGGTTACA	GAGAAGGGATGCGGTGGAA
Olig2	CTGGCGCGAAACTACATCC	CCCCGTAGATCTCGCTCAC

Opalin	GATCCAGCGAAGAAGAACCA	TGGGATTGTCATAGAGTTCTGAAA
Pcdh1	GAAGCCGAGCTGTCTGAGAA	GCACCTTGGTCTGAGTCGTTA
Pcdh9	TTAAGTTGGTGCCCCTCTCA	CTGCATTCATTCCGGTGTCA
Pcdhb11	TGGCCACCTGATGGATGTTA	CTCACCAGTCCTAGAGTCTCC
Pcdhb15	TGCTGCTGTTCGTTGGAGTA	GTGTTCTTCAGGCACAAAGCA
Pcdhb2	CAATGCCCCGGTGTTTTCAA	AGTAGCAACCTGGGATCCAAC
Pcdhb20	GGTGTGTTTCACAGGAGGTAC	ATCAGGGTCATGGAGCTGAA
Pcdhb22	TCGCTCACCAATCCAATCCC	TCCAGAGTCTCGGTCTCGAA
Pcdhga12	GAATGACAACGTCCCCGAAA	GTGCTAGCTCCACTCCAGTA
Pcdhga4	ATCCTGGCTGATCTGGTCAA	ACAGCTACCACCAGGTACAA
Pcdhga5	AGCCACTGTCACACTCACTA	CAGTGTGTTGTGGTTGCCTA
Pcdhga8	TGCAGACATGCTCATTAACCA	CTTGAGAGAAACGCCAGTCA
Pcdhgb1	GTGTTCAGCCAGGACACTTAC	GCTGTCACTTGCAGCACTAA
Pcdhgb4	TGAGCAGAACCCGGAATACA	AGGTGACACTTGAGCTGGAA
Pcdhgb8	GTGAGTGTCGCTGAGGAGAA	GGTCTCTGGGCTTGAGAGAAA
Pdgfra	CAAAGGGAGGACGTTCAAGAC	TGCGTCCATCTCCAGATTCA
Pja2	TGGCACTAGAAGAGCGCTTA	ATTGGCCACCTCAACGTCTA
Plp1	TGTGGACATGAAGCTCTCAC	AGAGGCAGTTCCATAGATGACA
Ptpre	CGATCCGAAAGTTCTGCATCC	GTGAAGTGCAGCTGTGAGAC
Scd1	AGGCCTGTACGGGATCATAC	AGCGCTGGTCATGTAGTAGAA
Sft2d2	GAAGCGCATGTTTGAGCCTA	CAGAACACAGGGTAAGTGCAAA
Shroom2	AAGTCCCCTTACCCAGAGTCA	AATTGGTGGCCAAGGACACA
Slc12a2	AGGAGCATTCAAGCACAGCTA	CCATATACAGAGCACTGGACACA
Slc12a6	GCATATGCGGCTGTCCAAAA	ATGCTGGTCAAGCGTAGCA
Slc22a23	CGCACTGGTCTCATCCAAAA	AGCGATATGGACTTTCCAGGTA
Slc27a1	GACCACCGGGCTTCCTA	CTGTAGGAATGGTGGCCAAA
Slc30a2	ACTGGTGGCAGCCTATATCA	CCCAAGACCAGGATGGAGAA
Slc30a7	CGGATTGGCAGCTTCTGTTA	AGCCAGGACTTCTGCTCTAA
Slc35e1	CCATTCTGGGTGTCTTCCTGTA	GTCTGACGTGGAGATGGGAA
Slc38a2	TTGCAGGCCACGCTATTTCA	CGGGATGGCAGACAAAGGAA
Slc39a9	GCCCAGCAGTTCCAAAATCA	TGCTGCTCCCAAAGCTACA
Slc5a3	CGCTACGAGTTGGCTTTGATT	GTTGCAGCTAAGTCTAGTGAAGC
Slc6a1	CTACCTGATTGGCCTGTCTAACA	TGCCGCTGGCAGAGTAATAA
Slc9a6	ACGCTGTTCACCTTCCAGAA	TGGCAGCTCTTCCCAAGAAA
Sox10	ACCAGTACCCTCACCTCCA	GCGCTTGTCACTTTCGTTCA
Sox5	CTCTGTCCCGGCAGCATTA	TCTTGGATTGCCTTGGTGACA
Sox6	TCTGGACTCAGCCCTTTACA	CATCTTTGCTCCAGGTGACA
Tcf7l2	GCACCGTAGGACAAATCCC	TCCTGTCGTGATTGGGTACA
Tet1	AGATGGCTCCAGTTGCTTATCA	ACGCCCCTCTTCATTTCCAA
Tet2	CTACATGGAGCTGAGCCAAA	TTGGAGCAATGACAGTAGCC
Tet3	ACGCCAGAGAAGATCAAGCA	GACAATCCACCCTTCAGAGACA
Thrap3	GAGGAAATGAACAAGAAGCAGCTAA	AGGTTCCCGTGGACTTTTCA
Tmed8	TGGCTTCTGCTTCTGATCCA	CCTTACTCGCTGGAGATACCA
Uhrf1bp1	TGGGACTCTGGCAGTTTCTAC	CCTGATGGGGTTGGTCTTCA

Usp6nl	TGGGCTCTTCTTCTTGAGATCC	ACCCCGTGCTCTGTGTTTTA
Wdr33	CAGGCACATAAGGAGGCGATTA	CCGTCATCAGAGCATGTAGCA
Wdr81	CGCCTGCTGACATCTTGTTA	CATTCAGTGGAGGGGTGTCA
Yipf6	CCTGTGGGGCCCATTGATAC	CCGCCATCATTCTTCCCATCA
Yy1	CAAGAACAATAGCTTGCCCTCA	GGTGTGCAGATGCTTTCTCA
Zbtb41	ATCCCGGTTTGCACGGTTA	TGAACTGACGACCACATATATCACA
Zdhhc2	CTGCCTTTTCATTGCTGCTACC	TTGGCTTGAGTATCAGGCAGAC
Zfp319	GGAATAAAGAGGCGGCAGAGAA	CAGGGTCCTGGCAATGCTAA
Zfp507	GGAACCACAGACGAGTCCATAA	GCTGCACACATACCCACAC
Zfp84	CCTGGATAGTGGAGCAAGCA	AGGCAAGAACTACCAACTCTTTCTA
Znfx1	CGGCTGGTAAAAGTGAACATCC	CAAAAGGCGGGCGATTTCA

Supplementary Table 2. List of primers used for the Biomark study.

Supplementary Table 3

Gene	Forward primer	Reverse primer
Gapdh	ACCCAGAAGACTGTGGATGG	CACATTGGGGGTAGGAACAC
Pja2	GCCTTGCCATCACTTCTTTC	GCAGATGCGTCAATAACTGC
Slc12a2	TTCCGCGTGAACTTCGTGG	TTGGTGTGGGTGTCATAGTAGT
Tet1	GAAGATAGTGTTCAGGGGGAAG	TACACGCTCACGAACCAAAC
Wdr33	TGATCTGGTCCCACCAATAG	TGACCAATCGTCTTCCTTCC

Supplementary Table 3. List of primers used for qRT-PCR.

Supplementary Figures:

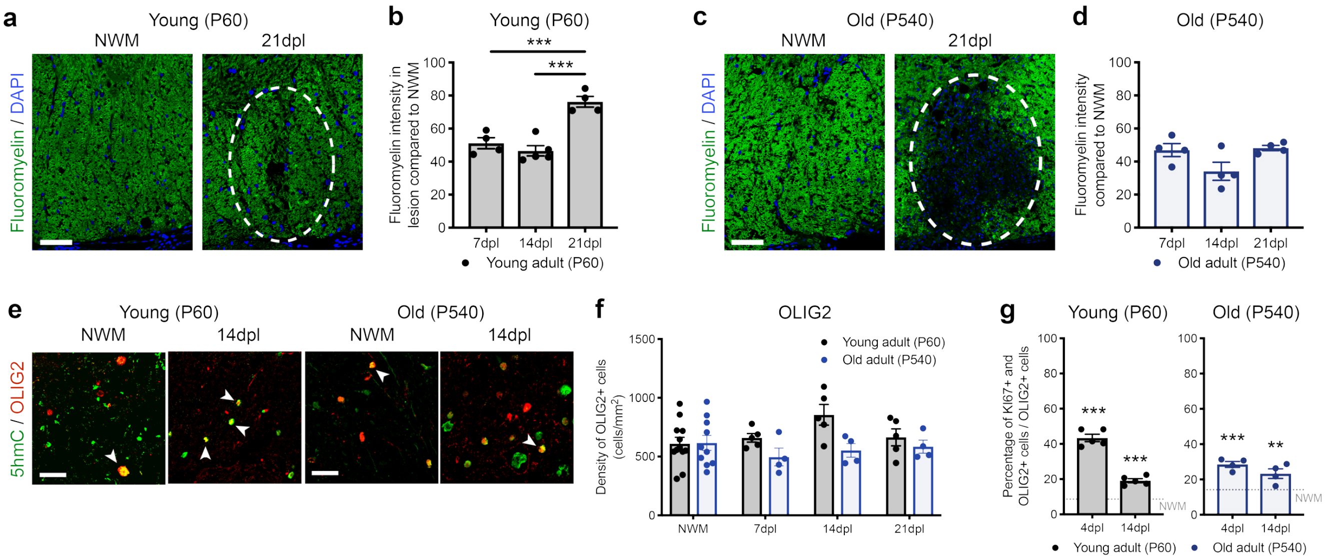

Supplementary Figure 1. Age-dependent decline of myelin repair after lesion.
(**a**) Representative confocal image of coronal spinal cord sections from young adult (P60) mice stained for Fluoromyelin (green) and DAPI (blue) as nuclear counterstain. Unlesioned normal white matter (NWM) is shown as comparison for the 21 days post lesion (21dpl) recovery after lysolecithin injection. The white dotted line indicates the average lesion size after lysolecithin injection. Scale bar = 100µm. (**b**) Quantification of Fluoromyelin intensity measured at the lesion side at several time points after injection (7dpl, 14dpl and 21dpl). Measurements are expressed as percentage, relative to the levels of intensity detected in the unlesioned white matter (NWM). Data represent average Fluoromyelin intensity measures in at least 4 sections/mouse in n=4 mice. Error bars represent SEM (***$p < 0.001$, One-way ANOVA for time). (**c**) Representative confocal image of coronal spinal cord sections from older mice (P540) mice stained for Fluoromyelin (green) and DAPI (blue) as nuclear counterstain. White dotted line identifies lesion site. Scale bar = 100µm. (**d**) Quantification of Fluoromyelin intensity measured at the lesion side in at least 4 sections/mouse in n=5 mice, at the indicated time points after injection. Values expressed as percentage, relative to the levels of intensity detected in the unlesioned white matter (NWM). Error bars represent SEM (ns, One-way ANOVA for time). (**e**) Confocal images of coronal spinal cord sections from young and old mice stained for 5hmC (green) in OLIG2+ (red). Representative stainings in unlesioned (NWM) and lesioned areas after 14 days from injection (14dpl) are shown. White arrowheads indicate OLIG2+ cells with high 5hmC immunoreactivity. Scale bar = 50µm. (**f**) Quantification of the density of OLIG2+ cells per mm^2 in unlesioned and lesioned areas of the spinal cord of young and old mice. Data represent average number of OLIG2+ cells per mm^2 ± SEM for n=4 young and n=5 old mice (ns, Two-way ANOVA, for age and time after lesion). (**g**) Quantification of the number of proliferating OLIG2+ cells identified by KI67 immunoreactivity at the indicated time points after lesion. Data are expressed as percentages in lesioned areas, compared to levels in NWM in young and old mice. The dotted line refers to the percentage of proliferating cells in unlesioned white matter at each age and serves as reference. Error bars represent SEM and values represent the average of 3 sections/mouse for n=4 young and n=5 old mice (**$p < 0.01$ and ***$p < 0.001$, One-way ANOVA, factor time).

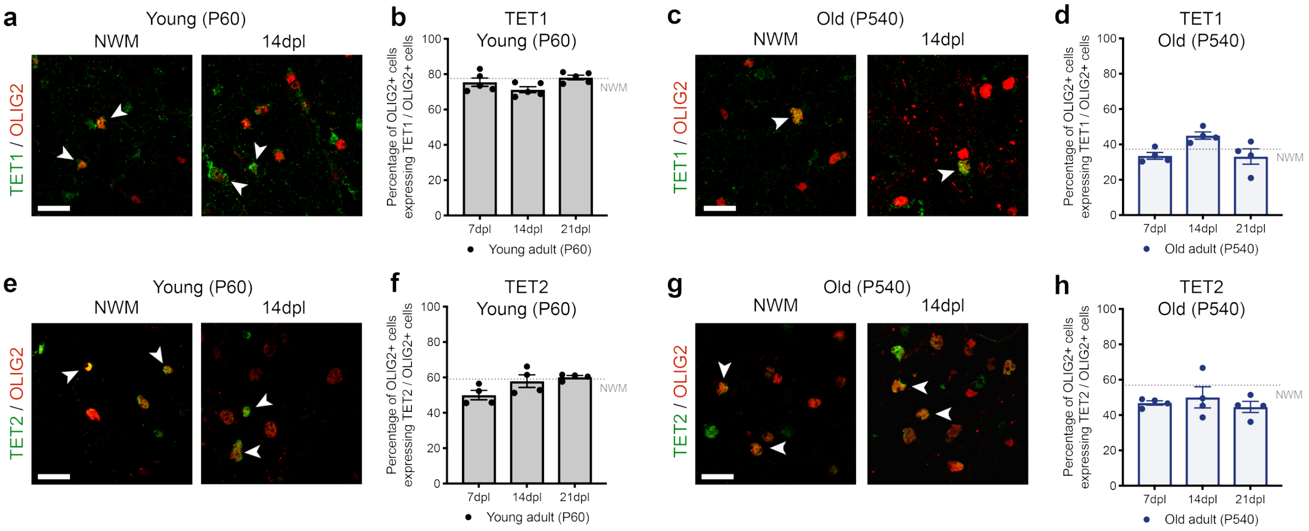

Supplementary Figure 2. Age-dependent decline of TET1 expression in OLIG2+ cells in the adult spinal cord during myelin repair after lysolecithin-induced demyelination.
(**a**) Representative confocal image of coronal spinal cord sections from young adult (P60) mice stained for TET1 (green) and OLIG2 (red). White arrowheads indicate co-labeled cells. Scale bar = 50μm. (**b**) Data represent the average percentage of TET1 expressing OLIG2+ cells relative to the total population of OLIG2+ cells, in lesioned areas of the spinal cord at 7dpl, 14dpl and 21dpl in young mice. Dotted line refers to the percentage of OLIG2+ cells expressing TET1 in unlesioned tracts. Error bars represent SEM, 3 sections/mouse, n=4 mice (ns, One-way ANOVA, factor time after lesion). (**c**) Representative confocal image of coronal spinal cord sections from old adult (P540) mice stained for TET1 (green) and OLIG2 (red). White arrowheads indicate co-labeled cells in unlesioned (NWM) and lesioned (14dpl) spinal cord. Scale bar = 50μm. (**d**) Data represent the average percentage of TET1 expressing OLIG2+ cells relative to the total population of OLIG2+ cells at the indicated time points in old mice. Dotted line refers to the percentage of OLIG2+ cells expressing TET1 in unlesioned tracts. The average was calculated by counting 3 sections/mouse in n=5 mice. Error bar represent SEM (ns, One-way ANOVA, factor time after lesion). (**e**) Representative confocal image of coronal spinal cord sections from young adult (P60) mice stained for TET2 (green) and OLIG2 (red). White arrowheads indicate co-labeled cells. Scale bar = 50μm. (**f**) Data represent the average percentage of TET2 expressing OLIG2+ cells relative to the total population of OLIG2+ cells, in lesioned areas of the spinal cord at 7dpl, 14dpl and 21dpl in young mice. Dotted line refers to the percentage of OLIG2+ cells expressing TET2 in unlesioned tracts. Error bars represent SEM, 3 sections/mouse, n=4 mice (ns, One-way ANOVA, factor time after lesion). (**g**) Representative confocal image of coronal spinal cord sections from old mice (P540) stained for TET2 (green) and OLIG2 (red). Scale bar = 50μm. (**h**) Data represent the average percentage of TET2 expressing OLIG2+ cells relative to the total population of OLIG2+ cells, in lesioned areas of the spinal cord at 7dpl, 14dpl and 21dpl in old mice. Dotted line refers to the percentage of OLIG2+ cells expressing TET2 in unlesioned tracts. The average was calculated by counting 3 sections/mouse in n=5 mice. Error bar represent SEM (ns, One-way ANOVA, factor time after lesion).

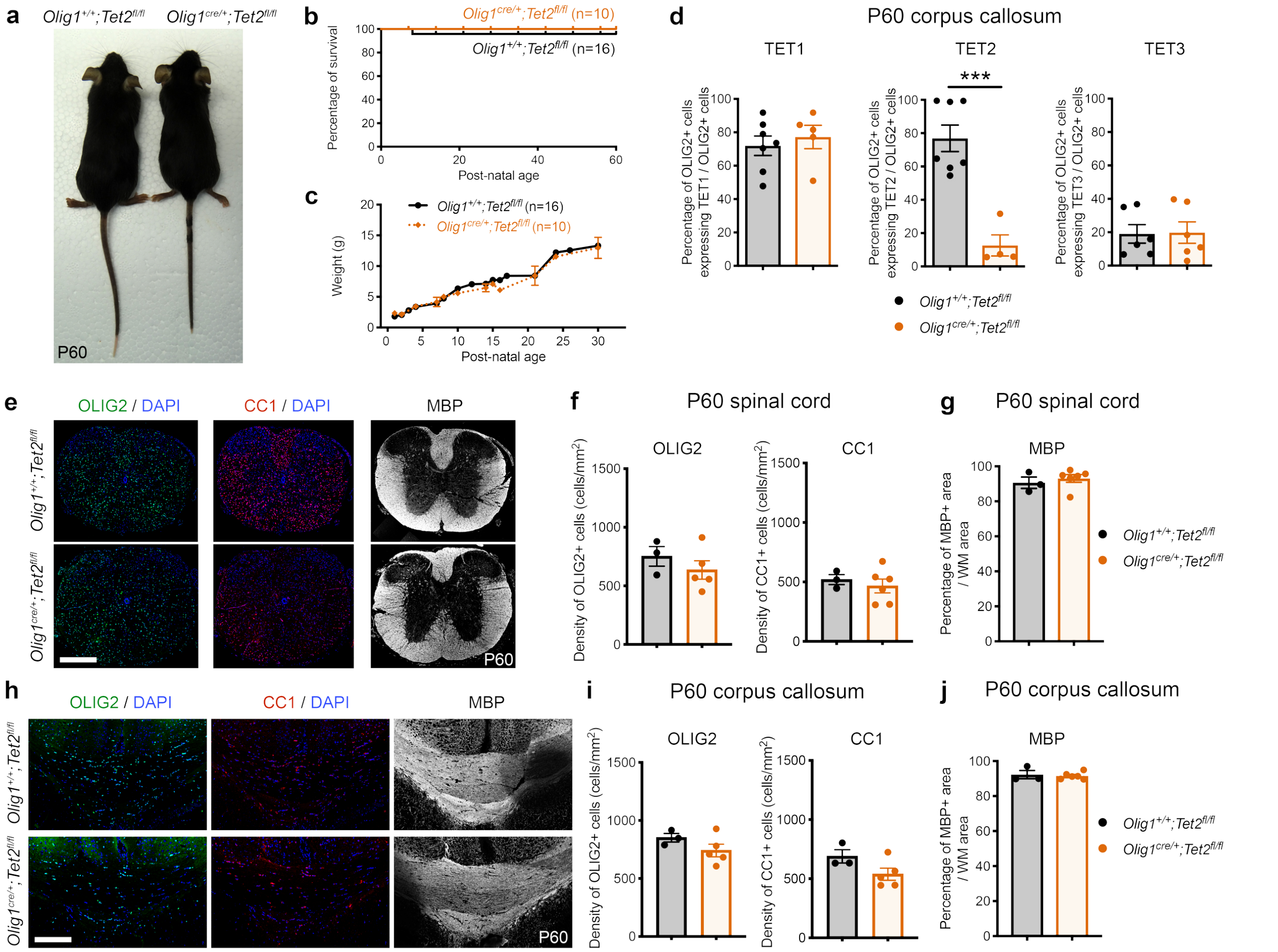

Supplementary Figure 3. Ablation of *Tet2* during development is compatible with normal myelination in the adult central nervous system.

(**a**) Photograph of young adult wild type (*Olig1*$^{+/+}$;*Tet2*$^{fl/fl}$) and Tet2 mutant (*Olig1*$^{cre/+}$;*Tet2*$^{fl/fl}$) mice at P60 reveals no difference in body size. (**b**) Kaplan–Meier survival curve and (**c**) body weight for *Olig1*$^{+/+}$;*Tet2*$^{fl/fl}$ (n=16) and *Olig1*$^{cre/+}$;*Tet2*$^{fl/fl}$ (n=10) mice. Error bars represent SEM. (**d**) Quantification of the percentage of OLIG2+ cells expressing TET1, TET2 or TET3 in the corpus callosum of P60 controls (*Olig1*$^{+/+}$;*Tet2*$^{fl/fl}$) and mutant (*Olig1*$^{cre/+}$;*Tet2*$^{fl/fl}$) mice. TET2 in oligodendroglial cells, without increased expression of TET1 or TET3. Data represent the percentage of TET-expressing OLIG2+ cells quantified in 3 sections/mouse in n=7 control mice and n=5 *Tet2* mutants. Error bars represent SEM (p=0.5762 for TET1, ***p=0.0004 for TET2, p=0.9300 for TET3, Student's t-test two-tailed). (**e**) Representative confocal images of P60 coronal spinal cord sections from controls and mutant mice stained for OLIG2 (green), CC1 (red), MBP (white), DAPI (blue) as nuclear counterstain. Scale bar = 500µm. (**f**) Quantification of OLIG2+ and CC1+ cell density in the white matter spinal cord of controls and mutant mice at P60. Data represent average numbers of OLIG2+ and CC1+ cells per mm^2 quantified in n=3 control mice and n=5 *Tet2* mutants. Error bars represent SEM. (p=0.3788 for OLIG2 and p=0.5677 for CC1, Student's test two-tailed). (**g**) Quantification of the average MBP+ area, relative to the total white matter area of coronal spinal cord sections. Error bars represent SEM for n=3 control mice and n=5 *Tet2* mutants (p=0.5512, Student's t-test two-tailed). (**h**) Representative confocal images of P60 corpus callosum in coronal brain sections from mice of the two genotypes, stained for OLIG2 (green), CC1 (red) and MBP (white). DAPI (blue) as nuclear counterstain. Scale bar = 500µm. (**i**) Quantification of OLIG2+ and CC1+ cell density in the corpus callosum of controls and mutant mice at P60 Data represent average numbers of OLIG2+ and CC1+ cells per mm^2 quantified in n=3 control mice and n=5 *Tet2* mutants. Error bars represent SEM (p=0.2043 for OLIG2, p=0.1082 for CC1, Student's t-test two-tailed). (**j**) Quantification of the average MBP+ stained area in the corpus callosum relative to the entire extent of the corpus callosum in each section. Error bars represent SEM for n=3 control mice and n=5 *Tet2* mutants (p=0.7161, Student's t-test two-tailed).

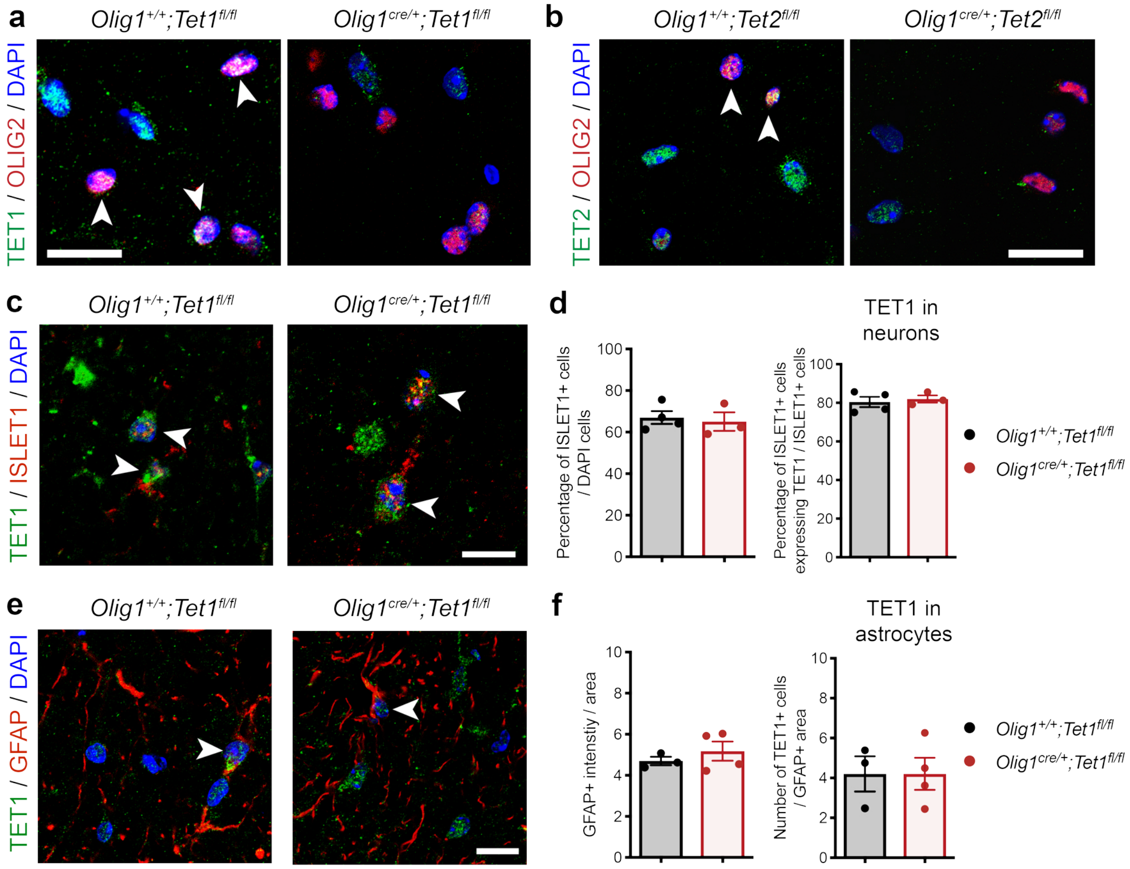

Supplementary Figure 4. Cell-specificity of *Tet1* ablation in *Olig1^{cre};Tet1$^{fl/fl}$* adult mice.
(**a**) Representative confocal images of P60 coronal spinal cord sections from control (*Olig1$^{+/+}$;Tet1$^{fl/fl}$*) or Tet1 mutant (*Olig1$^{cre/+}$;Tet1$^{fl/fl}$*) mice, stained for TET1 (green) and OLIG2 (red). DAPI (blue) as nuclear counterstain. White arrowheads indicate co-labeled cells. Scale bar = 50μm. (**b**) Representative confocal images of P60 coronal spinal cord sections from control (*Olig1$^{+/+}$;Tet2$^{fl/fl}$*) or Tet2 mutant (*Olig1$^{cre/+}$;Tet2$^{fl/fl}$*) mice, stained for TET2 (green), OLIG2 (red) and DAPI (blue). White arrowheads indicate co-labeled cells. Scale bar = 50μm. (**c**) Representative confocal images of P60 coronal spinal cord sections from control (*Olig1$^{+/+}$;Tet1$^{fl/fl}$*) or *Tet1* mutant (*Olig1$^{cre/+}$;Tet1$^{fl/fl}$*) mice, stained for TET1 (green) and the neuronal marker ISLET1 (red). White arrowheads indicate co-labeled cells. Scale bar = 50μm. (**d**) Bar graphs representing the percentage of ISLET1+ cells relative to total number of DAPI+ nuclei (left) and the percentage of TET1 expressing ISLET1+ cells in mice of the two genotypes (right). Error bar represent SEM for n=4 control mice and n=3 *Tet1* mutants (p=0.7223, p=0.6685, Student's t-test two-tailed). (**e**) Representative confocal images of P60 coronal sections from control (*Olig1$^{+/+}$;Tet1$^{fl/fl}$*) or Tet1 mutant (*Olig1$^{cre/+}$;Tet1$^{fl/fl}$*) mice, stained for TET1 (green), GFAP (red) and DAPI (blue). White arrowheads indicate co-labeled cells. Scale bar = 50μm. (**f**) Bar graphs represent the average intensity of GFAP+ per area in mice of the two genotypes (left) and the number of TET1 expressing cells per given GFAP+ area in controls and mutants (right). Error bar represent SEM for n=3 control mice and n=4 *Tet1* mutants (p=0.4448, p>0.9999, Student's t-test two-tailed).

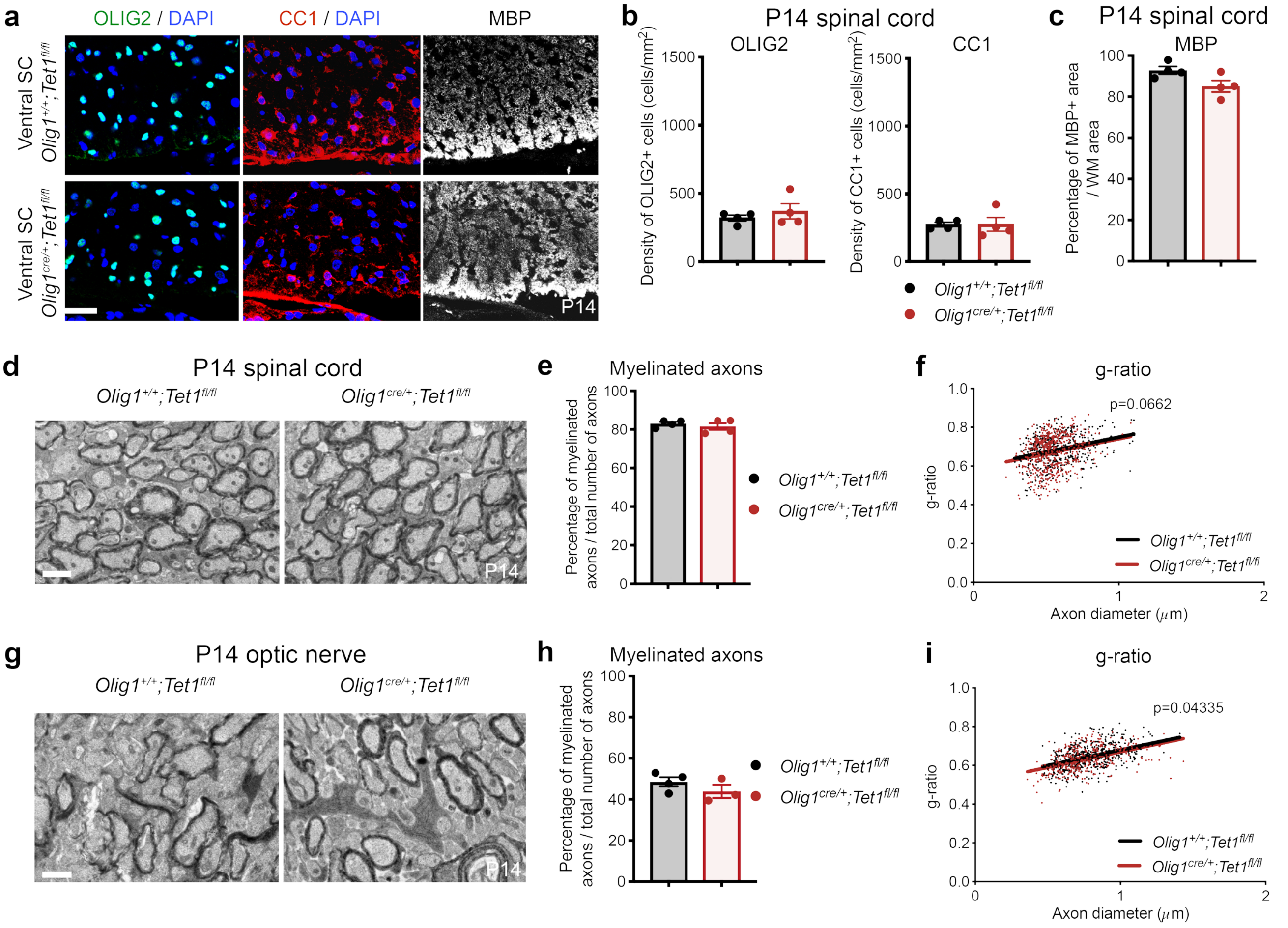

Supplementary Figure 5. Ablation of *Tet1* during development is compatible with normal myelination in the adult central nervous system.

(**a**) Representative high-magnification confocal micrograph of the ventral portion of the spinal cord in coronal sections from postnatal day 14 (P14) controls (*Olig1$^{+/+}$;Tet1$^{fl/fl}$*) and *Tet1* mutant (*Olig1$^{cre/+}$;Tet1$^{fl/fl}$* mice stained for OLIG2 (green), CC1 (red), MBP (white), DAPI (blue). Scale bar = 100μm. (**b**) Quantification of OLIG2+ and CC1+ cell density in mice of the indicated genotypes at the P14 developmental time point. Data represent average numbers of OLIG2+ or CC1+ cells per mm^2. Error bars represent SEM for n=4 mice (p=0.4507 for OLIG2, p=0.9815 for CC1, Student's t-test two-tailed). (**c**) Quantification of MBP+ area in the same section, referred as percentage of the total white matter (WM) region in the spinal cord. Error bar represent SEM for n=4 mice (p=0.0596, Student's t-test two-tailed). (**d**) Representative electron micrographs of myelinated axons in the P14 ventral white matter spinal cord from *Olig1$^{+/+}$;Tet1$^{fl/fl}$* and *Olig1$^{cre/+}$;Tet1$^{fl/fl}$*. Scale bar = 5μm. (**e**) Percentage of myelinated axons referred to the total number of axons per measured area. Error bars represent SEM for n=4 mice (p=0.2680, Student's t-test two-tailed). (**f**) Scatter plot of the g-ratio calculated in the spinal cord of P14 control (black circles) and *Tet1* mutants (red circles) and referred by axonal diameter. Quantification in 80-195 axons per mouse, for n=4 mice (non-linear regression, p=0.0662, comparison of slopes with sum-of-squares F Test). (**g**) Representative electron micrographs of myelinated axons in the P14 optic nerve from mice of the indicated genotype. Scale bar = 5μm. (**h**) Percentage of myelinated axons referred to the total number of axons per measured area. Error bars represent SEM for n=4 control mice and n=3 *Tet1* mutants (p=0.4832, Student's t-test two-tailed). (**i**) Scatter plot of the g-ratio calculated in the optic nerve of control (black circles) and *Tet1* mutant (red circles) mice and referred by axonal diameter. Quantification in 93-170 axons for mouse and n=4 control mice and n=3 *Tet1* mutants (non-linear regression, p=0.4335, comparison of slopes with sum-of-squares F Test).

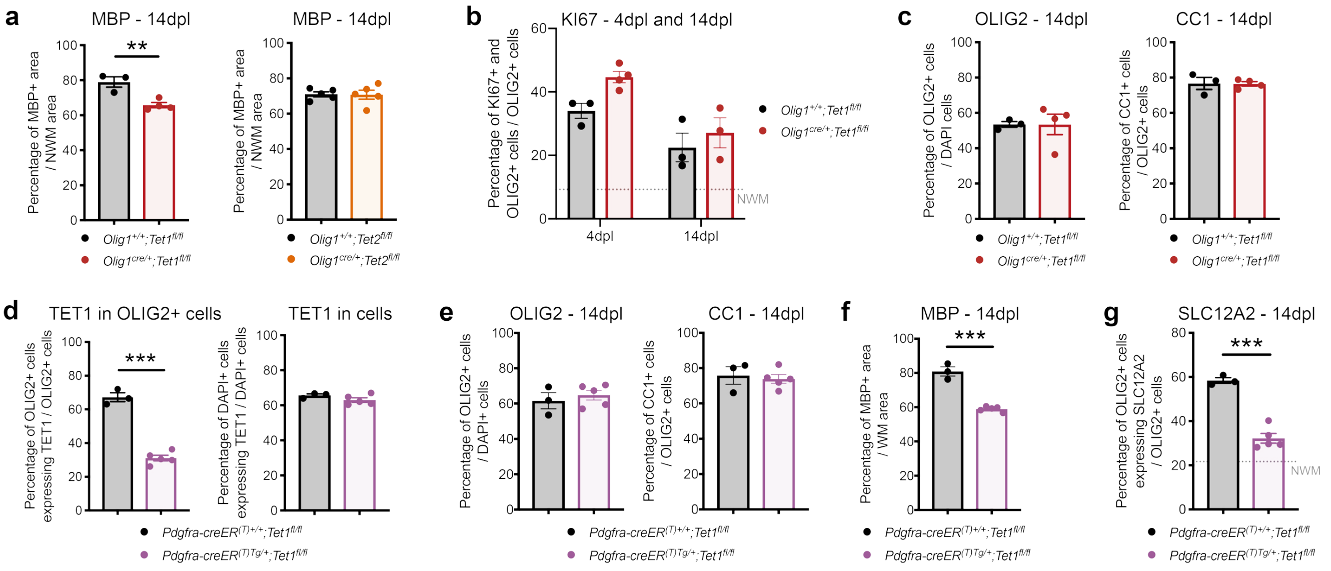

Supplementary Figure 6. Defective myelin repair in the spinal cord of adult mice with either constitutive or inducible ablation of *Tet1* in the oligodendrocyte lineage.

(**a**) Quantification of MBP$^+$ areas in the spinal cord of control *(Olig1$^{+/+}$;Tet1$^{fl/fl}$* and *Olig1$^{+/+}$;Tet2$^{fl/fl}$*), *Tet1* (*Olig1$^{cre/+}$;Tet1$^{fl/fl}$*) and *Tet2* mutant (*Olig1$^{cre/+}$;Tet2$^{fl/fl}$*) mice at 14 days post lesion. Data expressed as percentage of MBP+ area relative to the unlesioned white matter (NWM) area. Error bars represent SEM for n=3 control mice, n=4 *Tet1* mutants and n=5 control and *Tet2* mutants (**p=0.0075 for *Tet1* mutants, p=0.9388 for *Tet2* mutants, Student's t-test two-tailed). (**b**) Quantification of the percentage of proliferating OLIG2+ cells identified by KI67 immunoreactivity in an average of 3 spinal cord sections/mouse from 3 mice for each of the indicated genotypes at 4dpl and 14dpl. Dotted line refers to the percentage of proliferating OLIG2+ cells in unlesioned tracts (normal white matter, NWM). Error bars represent SEM for n=3 control mice and n=4 *Tet1* mutants (ns, Two-way ANOVA, for age and time after lesion). (**c**) Quantification of the percentage of OLIG2+ cells relative to total DAPI+ nuclei (left) and of the percentage of CC1+ cells relative to total OLIG2+ cells (right) in sections of the spinal cord from mice of the indicated genotypes, stained 14 days after lesion. Error bars represent SEM for n=3 control mice and n=4 *Tet1* mutants (p=0.9931 for OLIG2, p=0.9405 for CC1, Student's t-test two-tailed). (**d**) Quantification of TET1 expressing OLIG2+ cells in unlesioned controls (*Pdgfra-creER$^{(T)+/+}$;Tet1$^{fl/fl}$*) and inducible (*Pdgfra-creER$^{(T)Tg/+}$;Tet1$^{fl/fl}$*) mutant spinal cords, after tamoxifen induction. Data represent the average percentage of TET1 expressing cells relative to total OLIG2+ cells (left) and to DAPI+ nuclei (right). Error bars represent SEM for n=3 control mice and n=5 inducible *Tet1* mutants (***p < 0.0001 for OLIG2+ cells, p=0.1845 for DAPI+ cells, Student's t-test two-tailed). (**e**) Quantification of TET1 expressing OLIG2+ cells in lesioned controls (*Pdgfra-creER$^{(T)+/+}$;Tet1$^{fl/fl}$*) and inducible mutant (*Pdgfra-creER$^{(T)Tg/+}$;Tet1$^{fl/fl}$*) spinal cords, stained 14 days post lesion. Data represent average percentage of OLIG2+ cells relative to total DAPI+ nuclei (left) and of CC1+ cells relative to total OLIG2+ cells. Error bars represent SEM for n=3 control mice and n=5 inducible *Tet1* mutants (p=0.5478 for OLIG2, p=0.7003 for CC1, Student's t-test two-tailed). (**f**) Percentage of MBP+ area relative to total white matter (WM) in lesioned spinal cord assessed at 14dpl in mice of the indicated genotype, after tamoxifen induction. Error bars represent SEM for n=3 control mice and n=5 inducible *Tet1* mutants (***p < 0.0001, Student's t-test two-tailed). (**g**) Quantification of OLIG2+ cells also expressing SLC12A2 in lesioned spinal cord at the 14dpl time point in controls and inducible knockout mice, after tamoxifen gavage. Dotted line refers to the percentage of SLC12A2 expressing OLIG2+ cells in unlesioned tracts (NWM). Error bars: SEM for n=3 control mice and n=5 inducible *Tet1* mutants (***p=0.0001, Student's t-test two-tailed).

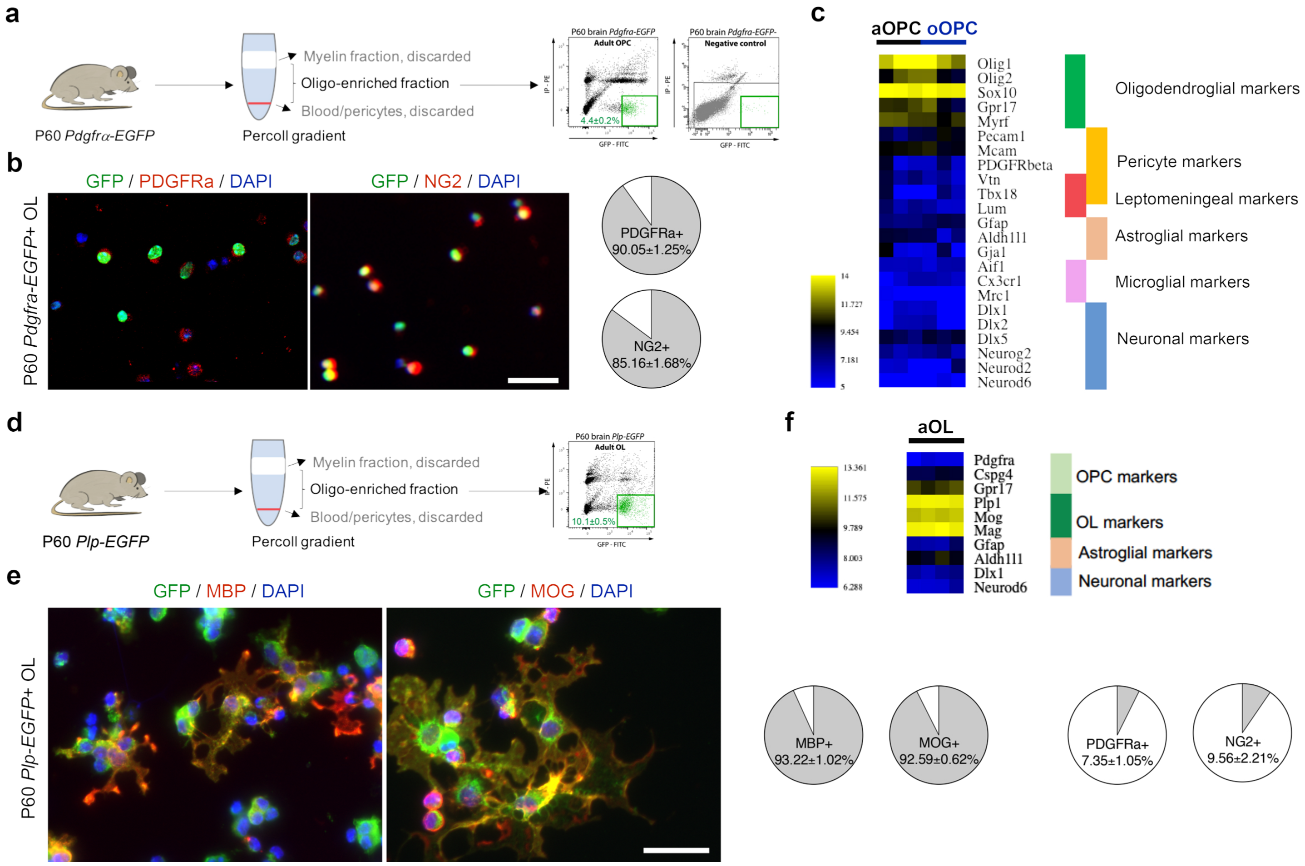

Supplementary Figure 7. Characterization of cells sorted from adult reporter mice.
(**a**) Adult (P60) *Pdgfra-H2BEGFP* reporter mice were used to perform a Percoll gradient isolation, in order to discard blood vessels and meninges and enrich for oligodendroglial lineage cells prior to fluorescence activated cell-sorting. (**b**) Immunofluorescence of sorted P60 *Pdgfra-H2BEGFP* cells (green), stained for the progenitor markers PDGFRα (red) or NG2 (red) 12 hours after plating. DAPI (blue) as nuclear counterstain. Scale bar = 100µm. Quantification from three independent experiments confirms that the majority of the sorted cells express the PDGFRα and NG2 markers. (**c**) Heatmap of the relative expression levels of the indicated cell-specific transcripts in three independent preparation of cells sorted from young adults (aOPC) or from P540 old mice (oOPC). Note the high expression of oligodendroglial lineage markers. (**d**) Adult (P60) *Plp-EGFP* reporter mice were used to perform a Percoll gradient isolation, in order to discard blood vessels and meninges and enrich for oligodendroglial lineage cells prior to fluorescence activated cell-sorting. (**e**) Immunocytochemistry of sorted P60 *Plp-EGFP* cells (green), stained for the mature markers MBP (red) and MOG (red). DAPI (blue) as nuclear counterstain. Scale bar = 100µm. Quantification from three independent experiments confirms that the majority of the *Plp-EGFP* sorted cells are mature OL, with high levels of MBP and MOG, and lacking the progenitor markers PDGFRα and NG2. (**f**) Heatmap of the relative expression levels of the indicated cell-specific transcripts in three independent preparation of P60 OL sorted from adult *Plp-EGFP* mice (aOL), identifying the enriched expression of myelin markers.

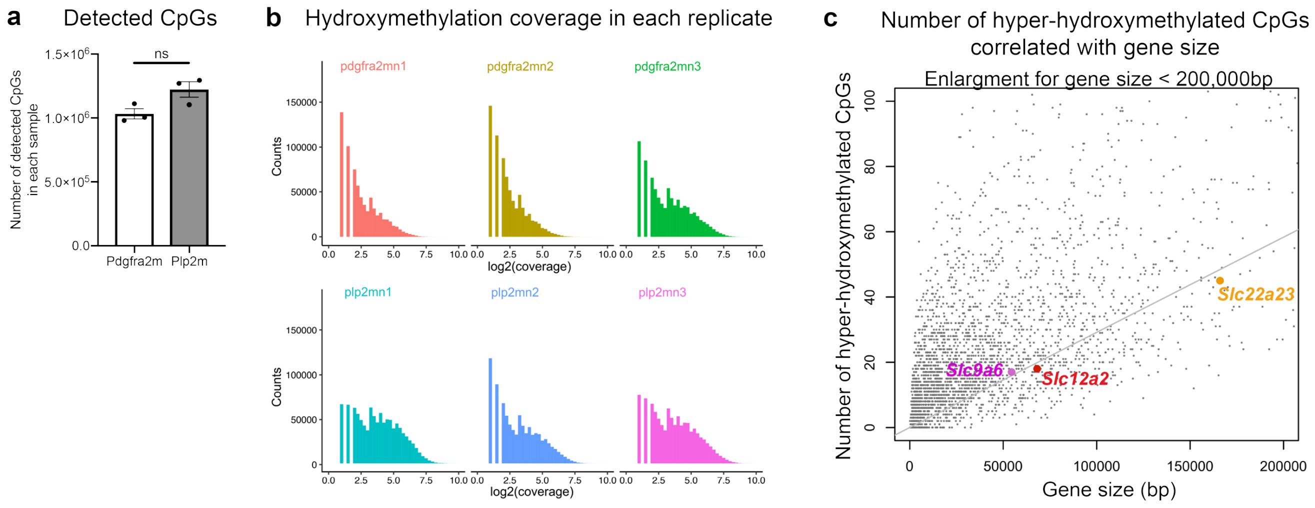

Supplementary Figure 8. Quality control of Reduced Representation Hydroxymethylation Profiling of adult progenitors and oligodendrocytes sorted from reporter mice.
(**a**) Average number of CpGs detected in each of the 6 independent biological samples identified by the by RRHP analysis. White bar graph refers to the number of CpGs in the aOPC samples prepared from sorted *Pdgfra-H2BEGFP* cells. Gray bar graph refers to number of CpGs in aOL sorted from *Plp-EGFP* mice. Error bars represent SEM for n=3 *Pdgfra-H2BEGFP* and n=3 *Plp-EGFP* replicates (p=0.0576, Student's t-test two-tailed). (**b**) Histograms showing the overall CpG hydroxymethylation coverage in each sample of aOPCs (pdgfra2mn1, mn2, mn3) and aOLs (plp2mn1, mn2, mn3). As expected, the shape of the curve reflecting the overall distribution differs between aOPCs and aOLs, with the latter showing higher levels of DNA hydroxymethylation. (**c**) Correlation plot between gene size and number of hyper-hydroxymethylated CpGs at gene body regions. All the differentially expressed genes (up- and down-regulated) are shown (gray). Three examples of TET1-target genes are shown: *Slc12a2* (red), *Slc9a6* (pink) and *Slc22a23* (orange) in the enlargement of hyper-hydroxymethylated CpGs for genes under 200,000bp. Linear regression curve is in grey.

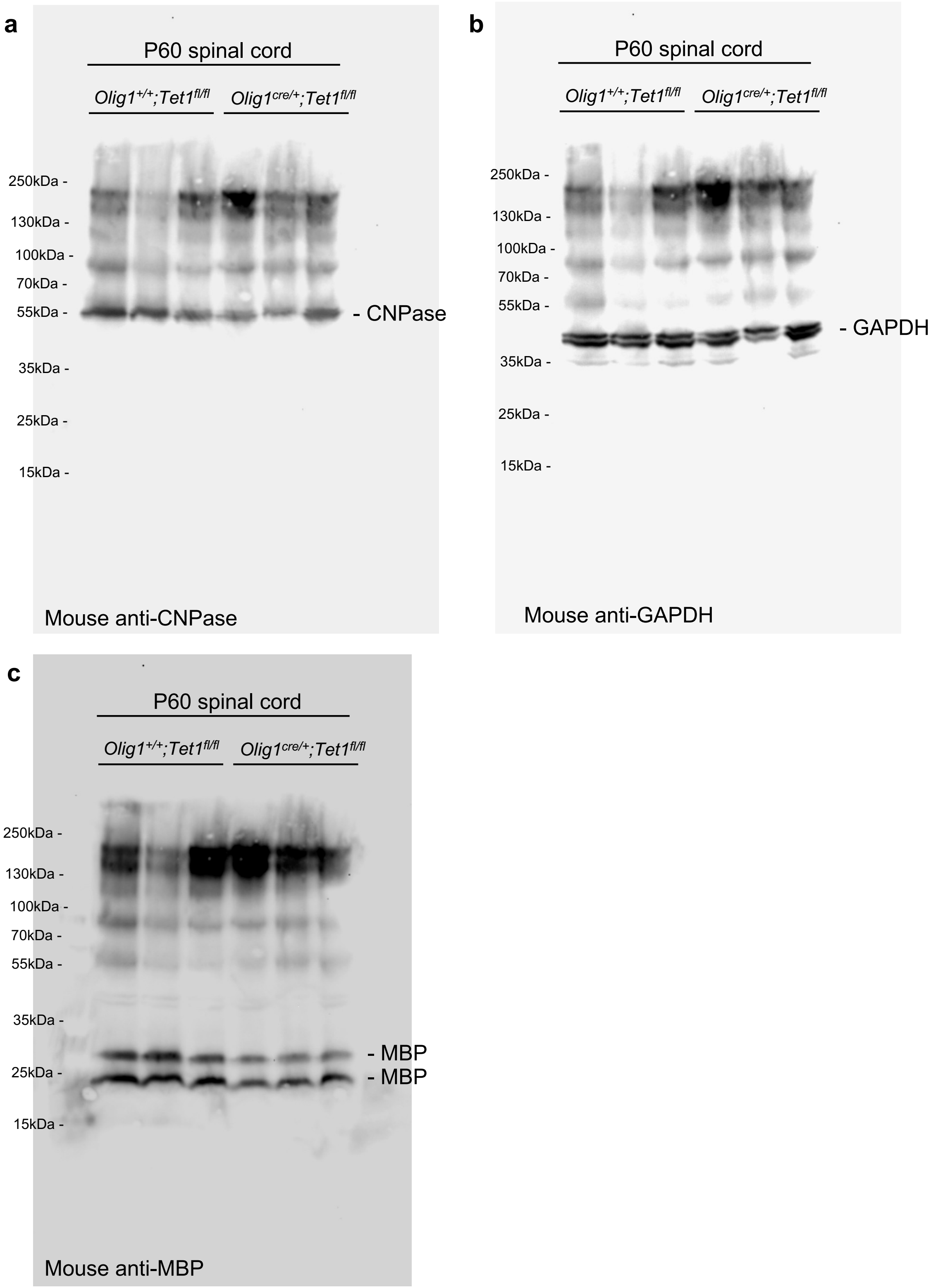

Supplementary Figure 9. Uncropped western blot gels.
(**a-c**) Full western blot of protein extract from P60 spinal cord of control (n=3) and *Tet1* mutants (n=3) stained for CNPase (**a**), GAPDH (**b**) and MBP (**c**). Molecular weight indicated on the left.
