# Supplementary material for: TET1-mediated DNA hydroxymethylation regulates adult remyelination in mice
Source: Nat Commun. 2021 Jun 7;12:3359. doi: 10.1038/s41467-021-23735-3 (PMC8185117; doi:10.1038/s41467-021-23735-3)
Supplement: Supplementary file 3 — Reporting Summary [file 41467_2021_23735_MOESM3_ESM.pdf]

nature research

Corresponding author(s): Patrizia Casaccia and Sarah Moyon

Last updated by author(s): Apr 15, 2021

Reporting Summary

Nature Research wishes to improve the reproducibility of the work that we publish. This form provides structure for consistency and transparency in reporting. For further information on Nature Research policies, see Authors & Referees and the Editorial Policy Checklist .

Statistics

For all statistical analyses, confirm that the following items are present in the figure legend, table legend, main text, or Methods section.

n/a	Confirmed	
☐	☒	The exact sample size (*n*) for each experimental group/condition, given as a discrete number and unit of measurement
☐	☒	A statement on whether measurements were taken from distinct samples or whether the same sample was measured repeatedly
☐	☒	The statistical test(s) used AND whether they are one- or two-sided<br>*Only common tests should be described solely by name; describe more complex techniques in the Methods section.*
☐	☒	A description of all covariates tested
☐	☒	A description of any assumptions or corrections, such as tests of normality and adjustment for multiple comparisons
☐	☒	A full description of the statistical parameters including central tendency (e.g. means) or other basic estimates (e.g. regression coefficient) AND variation (e.g. standard deviation) or associated estimates of uncertainty (e.g. confidence intervals)
☐	☒	For null hypothesis testing, the test statistic (e.g. *F*, *t*, *r*) with confidence intervals, effect sizes, degrees of freedom and *P* value noted<br>*Give P values as exact values whenever suitable.*
☒	☐	For Bayesian analysis, information on the choice of priors and Markov chain Monte Carlo settings
☒	☐	For hierarchical and complex designs, identification of the appropriate level for tests and full reporting of outcomes
☒	☐	Estimates of effect sizes (e.g. Cohen's *d*, Pearson's *r*), indicating how they were calculated

Our web collection on statistics for biologists contains articles on many of the points above.

Software and code

Policy information about availability of computer code

Data collection	Zen 2.3
Data analysis	R (4.0.3), Fiji Image J (2.1.0/1.53c), GraphPad Prism (9.0.1/128), Multi-experiment viewer (4.8.1)

For manuscripts utilizing custom algorithms or software that are central to the research but not yet described in published literature, software must be made available to editors/reviewers. We strongly encourage code deposition in a community repository (e.g. GitHub). See the Nature Research guidelines for submitting code & software for further information.

Data

Policy information about availability of data

All manuscripts must include a data availability statement. This statement should provide the following information, where applicable:
- Accession codes, unique identifiers, or web links for publicly available datasets
- A list of figures that have associated raw data
- A description of any restrictions on data availability

RNA sequencing and Reduced Representation Hydroxymethylation Profiling data from wild type adult mice have been deposited in NCBI's Gene Expression Omnibus Series accession number GSE122446. RNA sequencing from wild type and Tet1 mutants during myelin repair accession number GSE137611. Previously published and deposited data used in this study: GSE48872.

Field-specific reporting

Please select the one below that is the best fit for your research. If you are not sure, read the appropriate sections before making your selection.

Life sciences study design

All studies must disclose on these points even when the disclosure is negative.

Sample size	Sample size is indicated in the figure legend for each experiment. The sample size was chosen based on previous experience for each experiment to yield high power to detect specific effects. Previous publications on lysolecithin-lesions used a similar n=4-5 biological replicates for IHC and EM reproducibility (Fancy et al., 2011, Piaton et al., 2011). Previous publications on RNA-Sequencing for sorted pure populations used a similar n=3 biological replicates (Moyon et al., 2016, Segel et al., 2019).
Data exclusions	No data were excluded.
Replication	Attempts for replication were successful. For instance for P14 immunohistochemistry experiments, technical replicates were duplicated, evaluated by two independent investigators blind to genotype and replicated in independent biological samples. All rtqPCR and single-cell rtqPCR on Fluidigm/Biomark were all duplicated once on independent biological samples.
Randomization	Animals were chosen based on correct genotypes. Each experiment contained animals from at least two different litters to ensure that the differences can be observed in different litters. Within each group, all mice and cells were randomly selected and blindly evaluated
Blinding	All samples were attributed a single number/name during collection (perfusion, extraction). Investigators were blinded for IHC, EM and immunoEM acquisition. Even if blinded, investigators could notice IHC differences between neonatal (P7) and adult (P60) tissues, due to sample size. Investigators were blinded for quantification. Investigators were not blinded for cell-sorting (GFP marker needed to be known) and for RNA-Sequencing analysis (to be able to group replicates together).

Reporting for specific materials, systems and methods

We require information from authors about some types of materials, experimental systems and methods used in many studies. Here, indicate whether each material, system or method listed is relevant to your study. If you are not sure if a list item applies to your research, read the appropriate section before selecting a response.

Materials & experimental systems

n/a	Involved in the study	
☐	☒	Antibodies
☒	☐	Eukaryotic cell lines
☒	☐	Palaeontology
☐	☒	Animals and other organisms
☒	☐	Human research participants
☒	☐	Clinical data

Methods

n/a	Involved in the study	
☒	☐	ChIP-seq
☐	☒	Flow cytometry
☒	☐	MRI-based neuroimaging

Antibodies

Antibodies used

IHC: Fluoromyelin Green Fluorescent Myelin Stain (Invitrogen, F34651, 1:300), mouse anti-5mC (Abcam, ab10805, 1:200), rabbit anti-5hmC (Active Motif, 39769, 1:200), mouse anti-CC1 (Millipore, OP80, 1:200), rat anti-MBP (Abd Serotec, MCA095, 1:200), mouse anti-OLIG2 (Millipore, MABN50, 1:500), rabbit anti-OLIG2 (Santa Cruz, sc48817, 1:200), mouse anti-SLC12A2 (DSHB,T4, 1:100), rabbit anti-TET1 (Novus Biologicals, NBP1-78966, 1:100), rabbit anti-TET2 (Epigentek, A-1701, 1:100), rabbit anti-TET3 (Abcam, ab139311, 1:100), rabbit anti-KI67 (Abcam, ab21700, prediluted), mouse anti-GFAP (BioLegend, 644701, 1:200), mouse anti-ISLET1 (Abcam, ab86472, 1:200), secondary goat anti-mouse (IgG 488, Invitrogen, A11029, 1:1,000), secondary goat anti-rabbit (IgG 488, Invitrogen, A11034, 1:1,000), secondary goat anti-rabbit (IgG 546, Invitrogen, A11035, 1:1,000), secondary goat anti-mouse (IgG 546, Invitrogen, A11030, 1:,1000), secondary goat anti-rat (IgG 647, Life Technologies, A21247, 1:1,000).

ICC: rat anti-NFM-H (Millipore, MAB5448, 1:500), rabbit anti-NG2 (Millipore, AB5320, 1:200), mouse anti-SLC12A2 (DSHB, T4, 1:100), rat anti-PDGFRa (Millipore, CBL1366, 1:800), rat anti-MBP (Abd Serotec, MCA095, 1:200), mouse anti-MOG (Millipore, MAB5680, 1:500), mouse anti-OLIG2 (Millipore, MABN50, 1:200), rabbit anti-TET1 (Novus Biologicals, NBP1-78966, 1:100), secondary goat anti-rat (IgG 647, Life Technologies, A21247, 1:1,000), secondary goat anti-mouse (IgG 546, Invitrogen, A11030, 1:,1000), secondary goat anti-rabbit (IgG 488, Invitrogen, A11034, 1:1,000), secondary goat anti-rabbit (IgG 546, Invitrogen, A11035, 1:1,000), secondary goat anti-mouse (IgG 488, Invitrogen, A11029, 1:1,000), secondary goat anti-rat (IgG 555, Invitrogen, A21434, 1:1,000).

WB: mouse anti-CNPase (Sternberger Inc., SMI-91, 1:5,000), mouse anti-MBP (Millipore, SMI-99, 1:5,000), mouse anti-GAPDH (Abcam, ab8245, 1:5,000), secondary light-chain specific antibody goat-anti mouse IgG (Jackson Immunoresearch, 115-035-174, 1:10,000).

Immunopanning: rat anti-mouse CD140a antibody (BD Biosciences, 558774).

Validation	Antibodies have been validated by previous publications or by the company (https://www.abcam.com/5-methylcytosine-5-mc-antibody-33d3-ab10805.html, https://www.activemotif.com/catalog/details/39769, https://dshb.biology.uiowa.edu/T4, https://www.novusbio.com/products/tet1-antibody_nbp1-78966, https://www.epigentek.com/catalog/tet2-polyclonal-antibody-p-5015.html, https://www.abcam.com/tet3-antibody-ab139311.html, Moyon et al., 2015, 2016, 2017). Negative controls (without primary antibody) were used to assess the absence of non specific staining in immunohistochemistry, immunocytochemistry and immuno-EM experiments.

Animals and other organisms

Policy information about studies involving animals; ARRIVE guidelines recommended for reporting animal research

Laboratory animals	Mice were maintained in a temperature- (65-75F) and humidity- (40-60%) controlled facility on a 12-h light-dark cycle with food and water ad libitum. Mice from either sex were used and mutants were checked for survival and weight every day from birth to weaning.<br>Reporter lines: Pdgfra-H2BEGFP (RRID:IMSR-_JAX:007669), Plp-EGFP (Spassky et al, 1998).<br>Conditional knock-out mouselines: Olig1-cre (RRID:IMSR_JAX:011105), Tet1-flox (gift from. Pr. Yong-Hui Jiang) (Towers et al., 2018), Tet2-flox (RRID:IMSR_JAX:017573), Pdgfra-creERT (RRID:I MSR_JAX:018280).<br>Wildtype mice: C57bl6.<br>Adult zebrafish were housed and maintained in accordance with standard procedures in the Queen's Medical Research Institute zebrafish facility, University of Edinburgh. All experiments were performed in compliance with the UK Home Office, according to its regulations under project licenses 60/8436, 70/8436 and PP5258250. Adult zebrafish were subject to a 14/10 hours, light/dark cycle.<br>Zebrafish: Tg(mbp:EGFP-CAAX) transgenic line (Almeida et al, 2011).
Wild animals	No wild animals were used in this study.
Field-collected samples	No field-collected samples were used in this study.
Ethics oversight	All mouse experiments were performed according to IACUC-approved protocols. IACUC chaired by Dr. Dr. Steven Nicoll, members: Dr. Sulli Popilskis, Dr. Patrizia Casaccia, Dr. Jia Liu, Mr. Benjamin Inbar, Ms. Isela Lopez and Ms. Lesley Piccoli.

Note that full information on the approval of the study protocol must also be provided in the manuscript.

Flow Cytometry

Plots

Confirm that:

- [x] The axis labels state the marker and fluorochrome used (e.g. CD4-FITC).
- [x] The axis scales are clearly visible. Include numbers along axes only for bottom left plot of group (a 'group' is an analysis of identical markers).
- [x] All plots are contour plots with outliers or pseudocolor plots.
- [x] A numerical value for number of cells or percentage (with statistics) is provided.

Methodology

Sample preparation	Oligodendrocyte progenitor cells were isolated from post-natal day 5 (P5), P60 and P540 Pdgfra-H2BEGFP brains, oligodendrocytes from P16 and P60 Plp-EGFP brains, using fluorescence-activated cell sorting as described previously (Piaton et al, 2011 and Moyon et al, 2015). Tissue was dissected in HBSS 1X (HBSS 10X (Invitrogen), 0.01 M HEPES buffer, 0.75% sodium bicarbonate (Invitrogen), and 1% penicillin/streptomycin) and mechanically dissociated. After an enzymatic dissociation step using papain (30 ug/ml in DMEM-Glutamax, with 0.24 ug/ml L-cystein and 40 ug/ml DNase I), cells were put on a pre-formed Percoll density gradient before centrifugation for 15 min. Cells were then collected and stained with propidium iodide (PI) for 2 min at room temperature (RT). In a second step, GFP-positive and PI-negative cells were sorted by fluorescence-activated cell sorting (FACS; Aria, Beckton Dickinson) and collected in pure fetal bovine serum. Cells were washed twice in PBS 1X (PBS 10X, Invitrogen), then the dry cell pellets were frozen at -80°C.
Instrument	FACS; Aria, Becton Dickinson
Software	Cells were sorted using BD FACSDiva Software v8.0
Cell population abundance	Cell population abundance: P5 Pdgfra-H2BEGFP = 8.3±0.4%, P60 Pdgfra-H2BEGFP = 4.4±0.2%, P540 PPdgfra-H2BEGFP = 1.4 ±0.1%, P60 Plpl-EGFP = 10.1±0.5%,<br>Purity of each population has been previously characterized (Moyon et al., 2016) and detailed in Supplementary Fig.7 in this manuscript, using transcriptomic analysis and ICC data.
Gating strategy	Gating to isolate GFP+ cells has been set on GFP- lines, as negative control (example added in Supplementary Fig.7).

- [x] Tick this box to confirm that a figure exemplifying the gating strategy is provided in the Supplementary Information.
